# Silencing of a glycosyltransferase-like protein in citrus reduces male and female fertility impacting seed development in self-pollinated fruit

**DOI:** 10.3389/fpls.2025.1629727

**Published:** 2026-01-21

**Authors:** Stefania Bennici, Berta Alquézar, Lourdes Carmona, Gaetano Distefano, Alessandra Gentile, Leandro Peña

**Affiliations:** 1 Department of Agriculture, Food and Environment, University of Catania, Catania, Italy; 2 Instituto de Biología Molecular y Celular de Plantas, Consejo Superior de Investigaciones Científicas, Universidad Politécnica de Valencia, Valencia, Spain

**Keywords:** Carrizo citrange, FLOWERING LOCUS T, genetic transformation, RNA interference, seedlessness

## Abstract

*Citrus* species are among the most important fruit tree crops grown worldwide. Their long juvenile period joined with their complex genetic and reproductive characteristics severely hampers genomic studies and the improvement of traits of interest. Among these, seedlessness represents a major fruit quality trait. Genetic engineering is the fastest way to unequivocally characterize the function of citrus genes and to develop better varieties. In this study, two genes from *Citrus clementina* Hort. ex Tan., *CcGLT1* and *CcRBP1*, that putatively encode a glycosyltransferase-like (GLT) protein and an RNA binding (RBP) family protein, respectively, were characterized as highly expressed in male and female reproductive tissues and then evaluated as candidate genes involved in male and/or female gametic development by silencing them using RNA interference (RNAi) in Carrizo citrange, used as model citrus type easy to transform. Concurrently, the early flowering and fruiting phenotype was induced by ectopic overexpression of the citrus ortholog of the floral integrator *FLOWERING LOCUS T* gene (*FT*) which enabled flower and fruit production less than six months after transformation. Histological observations of flower tissues from genetically modified plants showed that silencing *CcGLT1* affects pollen performance by reducing pollen germinability and viability which results in an increased rate of ovule abortion resulting in fewer seeds in self-pollinated fruits. Conversely, the silencing of *CcRBP1* led to severe alterations in plant growth and development in the transgenic RBP lines preventing the characterization of its role in fertility, which therefore remains unresolved. These results provide useful insights into male and female sterility in citrus for the genetic improvement of commercial varieties aimed to obtain seedless fruits.

## 1 Introduction

Seedlessness is a major goal for breeding to improve fruit quality given the high demand from consumers for easy-to-eat fruits. It is a highly appreciated trait for both fresh consumption (e.g., banana, grape, citrus, watermelon) and processing (e.g., tomato sauces and fruit juices). Two main biological characteristics allow seedless fruit production: i) parthenocarpy, in which the ovary develops without fertilization resulting in fruits totally devoid of seeds or with an extremely reduced seed number (e.g., Corinth grapes, citrus, cucumber, tomato, pineapple); and ii) stenospermocarpy, in which pollination and fertilization are required for the ovary development but the ovule/embryo aborts resulting in fruits containing traces of the aborted seeds (e.g., grapes, watermelon) (Varoquaux et al., 2000; Picarella and Mazzucato, 2019). Several other genetic traits also contribute to the formation of seedless fruits such as female and/or male sterility (Cheng et al., 2023), pollination and fertilization failure due to self or cross-incompatibility (Wilcock and Neiland, 2002), defects in meiosis or endosperm development (Lora et al., 2011; Trojak-Goluch et al., 2021), and hormonal deregulations due to specific gene mutations (Varoquaux et al., 2000). In this sense, it is widely known that phytohormones play a key role in fruit set and seed development. A widespread agriculture practice to produce seedless parthenocarpic fruit consists in treating flowers with phytohormones before pollination. Increased auxin and gibberellin levels have been reported in pollinated ovaries of many fruit flowers (Pandolfini, 2009). The involvement of auxins in the induction of the parthenocarpy has been largely documented and it was suggested that this hormone replaces the signals provided by pollination and fertilization to initiate fruit growth in certain crops. Besides, on the other hand, the exogenous supply of GA_3_ was proposed to induce seedless fruit in cherimoya (Koura et al., 2004).

Citrus is one of the most economically important fruit crops worldwide, with more than of 166 million tons produced in 2022 (FAOSTAT, 2024). The increasing demand for high-quality fruit has made the development of seedless fruit cultivars a major goal for citrus fruit breeders, especially for mandarins. In citrus, several varieties such as those from the groups of Navel oranges, Satsuma mandarins and Clementine mandarins, among others, show the parthenocarpic ability to produce seedless fruit in the absence of fertilization (Mesejo et al., 2013). Combined with its parthenocarpic ability (Vardi et al., 2008; Aleza et al., 2010), seedlessness in citrus occurs naturally as a consequence of female and/or male sterility, embryo abortion and/or self-incompatibility (Sykes, 2008; Distefano et al., 2009; Qin et al., 2015; Goto et al., 2016; Bennici et al., 2019; Aloisi et al., 2020). Within fertile genotypes, it can be induced through gamma irradiation, which causes the occurrence of spontaneous mutations affecting male and female fecundity (Goldenberg et al., 2014; Huang et al., 2017; Kundu and Dubey, 2020; Cimen et al., 2021) or by ploidy manipulation, as triploid hybrids are usually sterile (Aleza et al., 2012, Aleza et al., 2016). Moreover, seedlessness may be affected by the environmental conditions as well as the application of agronomic techniques to reduce cross-pollination (Chao et al., 2005; Mesejo et al., 2006, Mesejo et al., 2008; Sykes, 2008; Gambetta et al., 2013).

In citrus, the molecular mechanisms underlying male and/or female sterility and ovule/seed development are poorly understood. Several comparative transcript profiling studies performed between seedy citrus genotypes and their seedless mutants revealed a range of genes with differential expression patterns associated with development, hormone and protein metabolism, gene regulation, signal cascades and nucleic acid binding that could be widely related with female and male sterility and seedless fruit development (Zheng et al., 2012; Zhang et al., 2017; Xiao et al., 2018). Several candidate genes for which transcript level differences may be important for seedless fruit development have been identified, but their possible involvement in male and/or female sterility has not been characterized so far.

Genetic engineering approaches have been tested to induce parthenocarpic fruit development. Among them, genes regulating auxin, cytokinin or gibberellins biosynthesis or metabolism from either bacterial or plant origin have been engineered under the control of ovule/seed specific promoters to confer parthenocarpy to crops including eggplant, tomato, raspberry and cucumber (Pandolfini, 2009). In other cases, specific toxic proteins or RNases have been overexpressed specifically in ovules or pollen to induce sterility (Cheng et al., 2023).

Genomic studies and the improvement of citrus traits by traditional breeding is a time-consuming process because of the long juvenile period of most citrus types which require 5–15 years to initiate flowering and fruiting. Besides, the complex genetics and reproductive biology of citrus, including high heterozygosity, cross and self-incompatibility and facultative apomixis, hampers even more the possibilities of classical breeding. Advances in genomics and biotechnology strategies provide useful resources for genetic studies and facilitate the selection in citrus breeding programs. In this study, we have used Carrizo citrange as a model citrus type because it is easy to transform genetically, and it is both self- and cross compatible rendering many seeds per fruit. In this study, an inspection of genome databases from proprietary seedless and seeded varieties (irradiated versus non-irradiated counterparts) revealed single-nucleotide polymorphisms (SNPs) in the coding sequence of some of the candidate genes from *C. clementina* to be investigated in detail with the aim to decipher their possible implications in fertility and to test them as targets to attempt inducing seedlessness in citrus. Additionally, a search focused on genes with preferential expression in pollen and/or ovule according to citrus transcriptome databases was performed. Two candidate genes were selected including *Ciclev10004681m* and *Ciclev10027731m* putatively encoding a glycosyltransferase-like protein (named *CcGLT1*) and an RNA binding (RRM/RBD/RNP motifs) family protein (named *CcRBP1*), respectively.

Here, we have investigated the expression profiles of *CcGLT1* and *CcRBP1* in leaf and flower tissues and, once confirmed their main expression in male and/or female reproductive tissues, we transformed plants to knock-down the expression of each of them or both together by RNA interference (RNAi). Concurrently, an early flowering and fruiting phenotype was induced by overexpression of the floral integrator *FLOWERING LOCUS T* gene from sweet orange (*CsFT*) to speed up phenotypic observations and characterization of transgenic plants and their fruits. *CcGLT1* is involved in pollen and ovule development in such a way that blocking its expression may cause sterility, and thus seedlessness upon-pollination.

## 2 Results

### 2.1 *CcGLT1* and *CcRBP1* contain pollen and seed-specific expression motifs in their promoter sequences and their expression patterns confirm that they are highly expressed in reproductive tissues

*C. clementina Ciclev10004681m*.*g* and *Ciclev10027731m.g* were selected as candidate genes considering the presence of SNPs in the genomic sequence of seedlessness mutants according to our genome databases (data not shown). Ciclev10004681m (XP_006421212.1) protein sequence showed the presence of conserved motifs characteristic of the Glycosyltransferase family 92 domain (IPR008166) (**Supplementary Figure S1A**) and therefore was named as Glycosyltransferase 1 (CcGLT1). Ciclev10027731m (XP_024035447.1) protein sequence showed the presence of conserved motifs characteristic of the family to which it belongs, such as the RNA recognition motif domain (IPR000504) and Zinc finger, RING-type (IPR001841) (**Supplementary Figure S1B**) and therefore named as RNA binding protein family 1 (CcRBP1).

**Figure 1:**
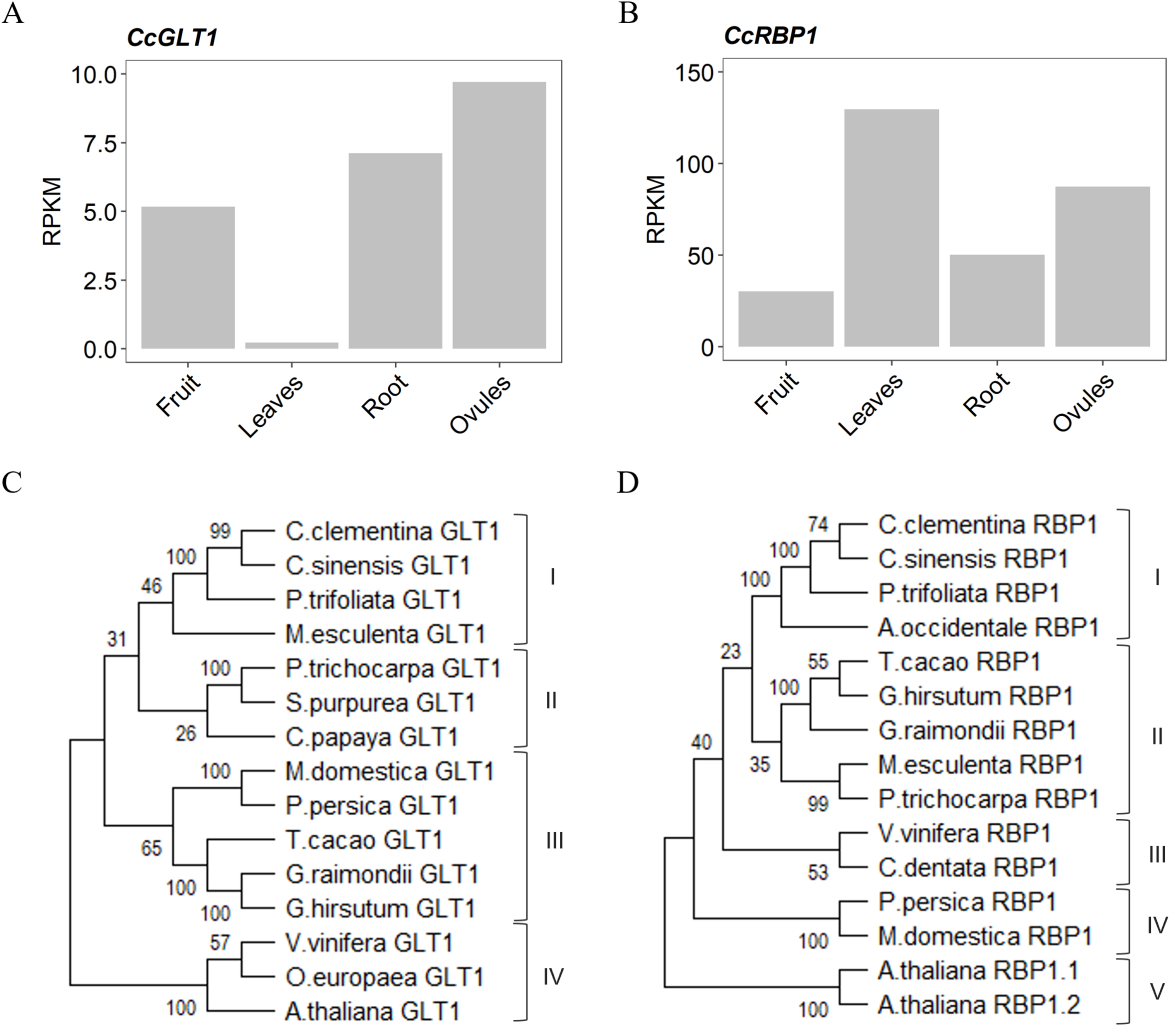
Reads per kilobase per million mapped reads (RPKM) values in fruit, leaves, root and ovules for CcGLT1 **(A)** and CcRBP1 **(B)**, according to RNA-seq data from Citrus Annotation Project database (CAP). **(C)** Phylogenetic tree of glycosyltransferase-like proteins (GLT1) encoded by *Citrus clementina* (Ciclev10004681m.g), *Citrus sinensis* (Cs_ont_9g017790.1), *Poncirus trifoliata* (Ptrif.0009s1448), *Manihot esculenta* (Manes.09G016100), *Populus trichocarpa* (Potri.016G041900), *Salix purpurea* (SapurV1A.0018s0900), *Carica papaya* (evm.TU.supercontig_120.11), *Malus domestica* (MD12G1085800), *Prunus persica* (Prupe.7G049900), *Theobroma cacao* (Thecc.K0006500), *Gossypium raimondii* (Gorai.007G355500), *Gossypium hirsutum* (Gohir.D11G324700), *Vitis vinifera* (VIT_213s0019g01720), *Olea europaea* (Oeu027348.1), and *Arabidopsis thaliana* (AT3G08550). **(D)** Phylogenetic tree of RNA binding (RRM/RBD/RNP motifs) family proteins (RBP1) encoded by *Citrus clementina* (Ciclev10027731m.g), *Citrus sinensis* (Cs_ont_8g004030), *Poncirus trifoliata* (Pt8g008970.1), *Anacardium occidentale* (Anaoc.0005s1250), *Theobroma cacao* (Thecc.09G336200), *Gossypium hirsutum* (Gohir.A07G093300), *Gossypium raimondii* (Gorai.004G002000), *Manihot esculenta* (Manes.04G026900), *Populus trichocarpa* (Potri.009G026200), *Vitis vinifera* (VIT_206s0004g03580), *Castanea dentata* (Caden.08G041800), *Prunus persica* (Prupe.6G091300), *Malus domestica* (MD03G1108000), *Arabidopsis thaliana 1* (AT3G45630), *Arabidopsis thaliana 2* (AT5G60170). The dendrograms were generated with MEGA11 using the Neighbor-joining method.

Both candidate genes were also selected for their prevailing expression in male and female tissues according to Citrus Annotation Project (CAP, http://citrus.hzau.edu.cn/orange/) database (**Figures 1A, B**). *In silico* gene expression analysis showed that *CcGLT1* transcription is much higher in ovules than in other tissues (**Figure 1A**). Similarly, high accumulation in ovules was found for *CcRBP1* transcript (**Figure 1B**) together with a much higher accumulation in leaves (**Figure 1B**).

To explore the diversification history of the candidate proteins in relation to other dicotyledonous plant orthologs, a phylogenetic tree was constructed for each of them. Results revealed four main groups of GLT1 (**Figure 1C**): one group consists of GLT1 proteins from sweet orange and trifoliate orange, both parents of citrange, showing a strong closeness with CcGLT1 and a protein of *Manihot esculenta*; the second group contains proteins from two members of the Salicaceae family (*Populus trichocarpa* and *Salix purpurea*) and *Carica papaya*; the third group contains proteins from two members of the Rosaceae family (*Malus domestica* and *Prunus persica*), two members of Malvaceae family (*Gossypium raimondii* and *G. hirsutum*) and *Theobroma cacao*; the fourth group, showing a greater distance from the proteins of citrus (group I, **Figure 1C**), contains proteins from *Arabidopsis thaliana, Vitis vinifera* and *Olea europaea*. Phylogeny analysis for CcRBP1 revealed five main groups (**Figure 1D**): the first group includes proteins from Clementine, trifoliate and sweet orange, all showing a great closeness, and a protein from *Anacardium occidentale*; the second group contains proteins from *T. cacao*, *M. esculenta*, *P. trichocarpa* and two members of genus *Gossypium* (*G. hirsutum* and *G. raimondii*); the third group includes two proteins of *Vitis vinifera* and *Castanea dentata*; the fourth group contains the proteins of Rosaceae *P. persica* and *M. domestica;* the fifth group, the farthest from citrus (group I, **Figure 1D**), contains proteins of *A. thaliana* (RBP1.1 and RBP1.2).

To further explore the envisaged transcription of *CcGLT1* and *CcRBP1* in reproductive tissues, the 2-kb upstream sequence of both genes was analyzed *in silico* and compared to those of other plant orthologs (**Supplementary Figure S2A**). Results revealed the presence of *cis*-acting elements along 700-bp upstream of the coding sequence of both genes that are reported to be involved in pollen and seed expression (**Supplementary Figure S2B**).

**Figure 2:**
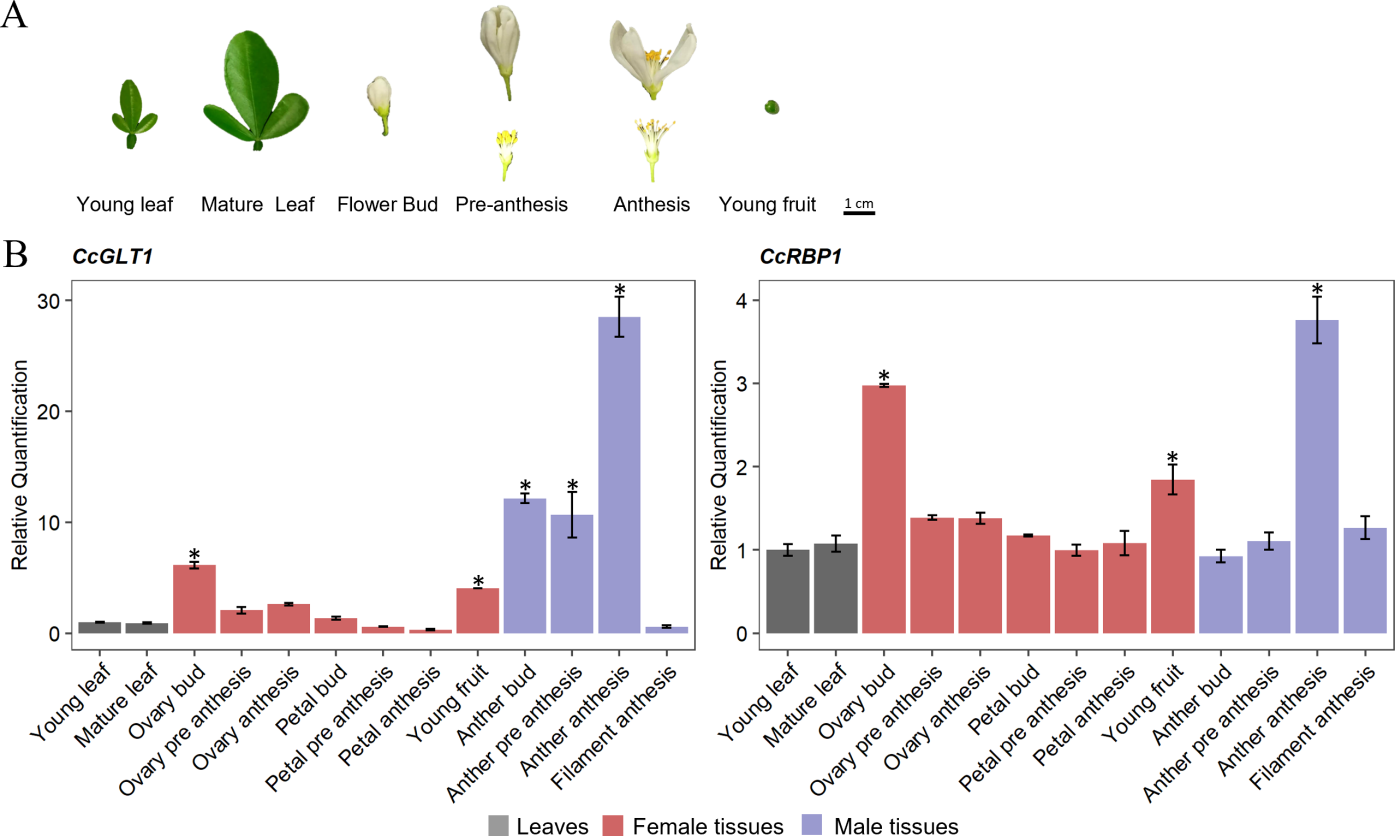
**(A)** Starting material used for RNA extraction to study expression of *CcGLT1* and *CcRBP1* genes in leaves and different male and female reproductive tissues of Carrizo citrange, Young leaf, 2 cm long; Mature leaf, 3 cm long; ovary and anthers collected from Flower buds, 1 cm long, Flower at pre-anthesis and Flower at anthesis; Young fruit, 5 mm long. Scale bar: 1 cm. **(B)** Relative quantification of expression of *CcGLT1* and *CcRBP1* genes in different tissues shown in **Figure 1A**. Data are presented as the mean relative expression ± SD of each individual sample as compared to the control sample (Young Leaf). Statistical analyses were performed using analysis of variance (ANOVA) and an asterisk above the bars indicates significantly different values at *p*-value < 0.01 (*).

Thus, the *in silico* expression data and the presence of *cis*-acting regulatory sequences specific for reproductive tissues encouraged us to further characterize *CcGLT1* and *CcRBP1* transcription patterns. Expression of candidate genes *CcGLT1* and *CcRBP1* was assayed by qRT-PCR analysis in different tissues including leaves at two developmental stages (young and mature), ovaries and anthers from flower buds, flowers at pre-anthesis and anthesis and young fruits from Carrizo citrange (**Figure 2**). Results showed that in female tissues both *CcGLT1* and *CcRBP1* transcripts accumulated in the ovary during the flower bud stage and that their expression was higher than in ovaries from later developmental stages (three and two-fold increased respect ovary during pre-anthesis and at anthesis, respectively for *CcGLT1;* two-fold increased for *CcRBP1*) and also higher than in petals (five and three-fold higher for *CcGLT1* and *CcRBP1*, respectively) or leaves (six and three-fold higher for *CcGLT1* and *CcRBP1*, respectively) (**Figure 2B**). In male tissues, both *CcGLT1* and *CcRBP1* transcripts accumulated at higher levels in anthers during the anthesis stage than in anthers from previous developmental stages (two and three-fold increase respect to anthers during flower bud and pre anthesis stages, respectively for *CcGLT1* and four and three-fold increase compared to anthers during flower bud and pre anthesis stages, respectively for *CcRBP1*) (**Figure 2B**).

### 2.2 Carrizo citrange transformants show early flowering and fruiting associated to *CsFT* overexpression as well as RNAi-induced downregulation of *CcGLT1* and *CcRBP1* genes in reproductive tissues

**Figure 3:**
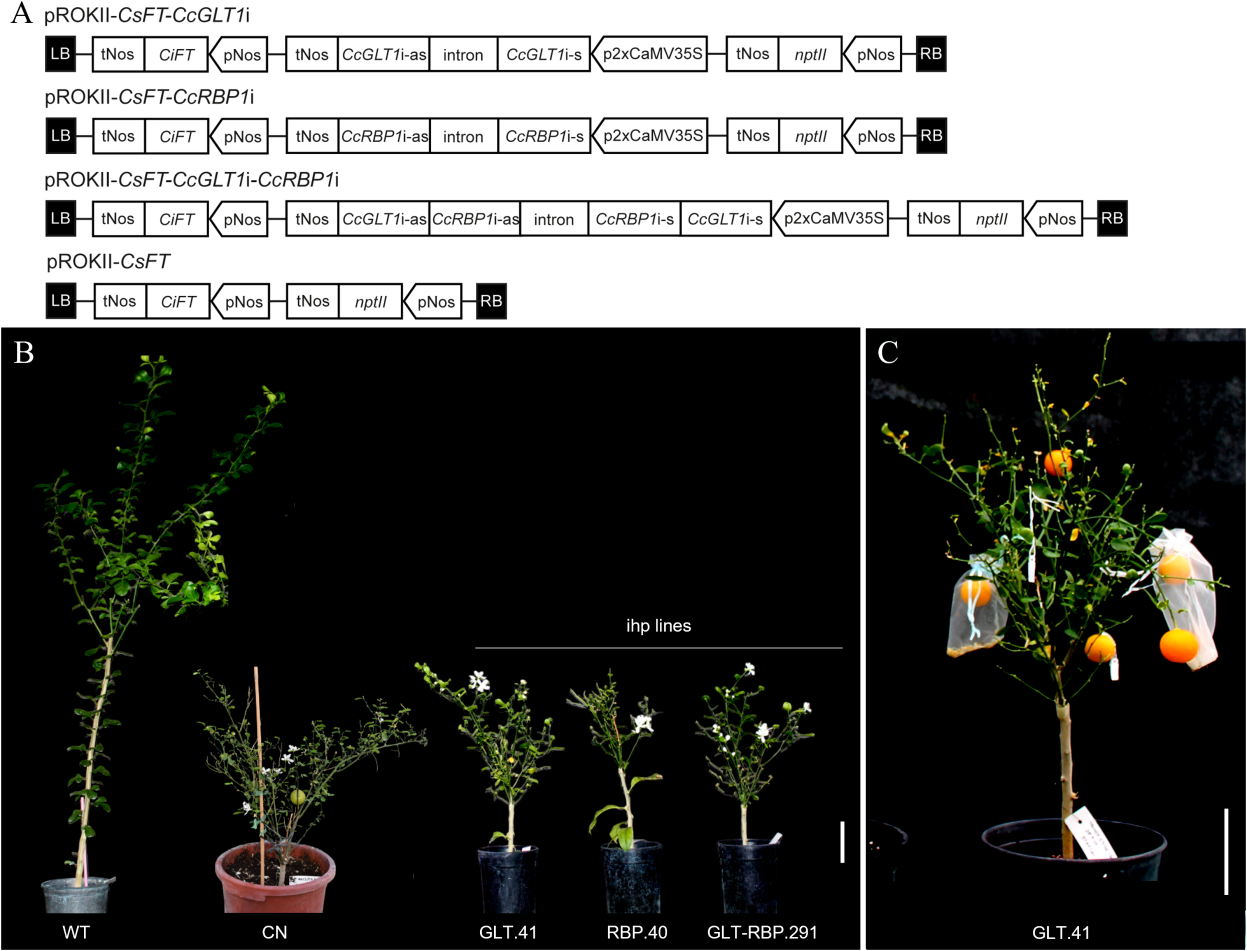
**(A)** Schematic representation of the T-DNA region from plasmids used for plant transformation. pROKII-CsFT-CcGLTi plasmid containing both an intron-hairpin (ihp) RNAi-triggering construct for silencing the expression of the *CcGLT1* gene and an CsFT overexpression cassette; pROKII-CsFT-CcRBPi plasmid containing both an ihp RNAi-triggering construct for *CcRBP1* gene silencing and an CsFT overexpression cassette; pROKII-CsFT-CcGLTi-CcRBPi plasmid containing both an ihp RNAi-triggering construct for *CcGLT1* and *CcRBP1* genes silencing and an CsFT overexpression cassette; pROKII-CsFT containing just the CsFT overexpression cassette used to transform control plants. pNos and tNos, the nopaline synthase gene promoter and terminator sequences; *nptII*, neomycin phosphotransferase II gene, conferring resistance to the antibiotic kanamycin; *CsFT*, FLOWERING LOCUS T from sweet orange; 2xCaMV35S, promoter of the 35S gene of the Cauliflower mosaic virus with duplicated enhancer sequence; CcGLT-s and CcGLTi-as, sense- and antisense-oriented sequences, respectively, designed to silence the expression of the *CcGLT1* gene; CcRBP-s and CcRBP-as, sense- and antisense-oriented sequences, respectively, designed to silence the expression of the *CcRBP1* gene; SL intron, intron sequence from *S. lycopersicum*; LB and RB, left and right borders of the T-DNA region. **(B)** Induction of early flowering in transgenic Carrizo citrange plants containing a citrus FLOWERING LOCUS T (*CsFT*) overexpression cassette. One CN line and one of each GLT, RBP and GLT-RBP lines, all carrying the *CsFT* transgene, exhibiting the early-flowering phenotype compared with the WT control. The photograph was taken 6 months after grafting in the greenhouse. **(C)** A representative plant with fruits at the full-colored stage 12 months after grafting in the greenhouse. Scale bar: 10 cm.

Characterization of selected candidate genes was attempted using a biotechnological approach that combines their silencing by RNAi and the concurrent overexpression of *CsFT* gene to induce early flowering and fruiting phenotype and thus speed up the characterization of transgenic fruit (Pons et al., 2014). Three plasmids containing both an *CsFT* overexpression cassette, the *nptII* selectable marker cassette and an intron-hairpin (ihp) cassette to silence either *CcGLT1*, *CcRBP1* or both genes, were generated (**Figure 3A**) and used to transform epicotyl segments of Carrizo citrange via *Agrobacterium tumefaciens-*mediated transformation. The plasmid pROKII-*CsFT* (**Figure 3A**) previously generated (Pons et al., 2014) was used to transform control (CN) plants. Kanamycin-resistant regenerants obtained after transformation were screened by PCR to verify the integration of the different T-DNAs using primers specific to the *CsFT* and *nptII* transgenes as well as to each ihp cassette (**Supplementary Table S1**). PCR-positive shoots, designated as CN (control plants transformed with pROKII-*CsFT*), GLT (plants transformed with pROKII-*CsFT*-*CcGLT*i), RBP (plants transformed with pROKII-*CsFT*-*CcRBP*i) and GLT-RBP (plants transformed with pROKII-CsFT-*CcGLT*i-*CcRBP*i) lines, were grafted onto vigorous citrus rootstocks in a greenhouse. Eight lines for each construct were obtained. *CsFT* overexpression caused a clearly visible early flowering phenotype, with most transgenic lines starting to flower during *in vitro* regeneration and again when grafted on vigorous rootstocks in the greenhouse, about 3 months after the transformation experiments, mainly exhibiting a terminal flower bud indicating that the branch had transitioned to flowering. Six months after grafting in the greenhouse, transgenic plants reached full flowering (**Figure 3B**, CN and ihp lines) and about six months later they reached the full fruiting stage (**Figure 3C**). Early flowering and fruiting phenotype confirmed the overexpression of the *CsFT* cassette in the transgenic lines.

Differences were observed in plant size and architecture among transgenic lines when compared to the non-transformed control plants (WT) because of the effect of *CsFT* overexpression in plant architecture. No significant difference was found between CN and GLT plants in relation to plant architecture, evaluated by measuring the branch length/number of nodes per plant. Conversely, RBP and GLT-RBP plants showed reduced sizes when compared to the other groups, CN and GLT (**Supplementary Figure S3**), indicating that downregulation of *CcRBP1* was likely affecting plant development, further compromising growth. RBP plants with a strong-reduced size were removed from the analysis and were evaluated plants for the *CcGLT1* gene knocking-down.

For the characterization of *CcGLT* RNAi and its effect on reproductive development, three plants were selected among GLT and GLT-RBP lines, based on their similar architectural phenotype, meaning that they were showing early flowering and fruiting, but their vegetative development was not compromised excessively. The selected transgenic plants included: GLT.41, GLT.73 and GLT.115 for GLT; GLT-RBP.15, GLT-RBP.71 and GLT-RBP.291 for GLT-RBP.

**Figure 4:**
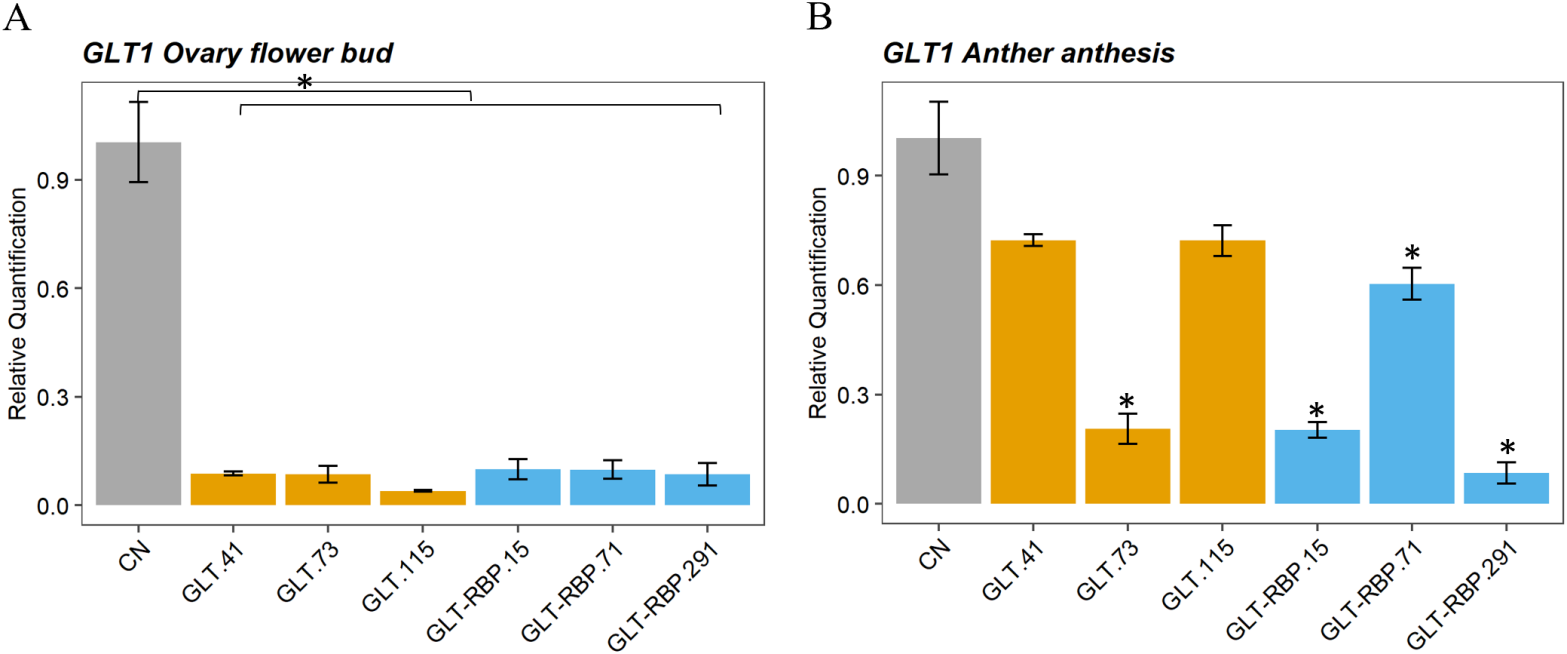
Relative quantification of expression of *CcGLT1* gene in ovary during flower bud stage **(A)** and anthers from flowers at anthesis **(B)** of transgenic GLT and GLT-RBP lines. Data are presented as the mean relative expression ± SD of each sample. cDNA from the control CsFT lines (CN) was used as a calibrator sample, and the rest of the values were expressed relative to this sample’s value. Statistical analyses were performed using analysis of variance (ANOVA) and an asterisk above the bars indicates significantly different values at *p*-value < 0.01 (*).

Knocking-down of the *GLT1* gene in GLT and GLT-RBP lines was evaluated by measuring endogenous *GLT1* transcript abundance in ovaries from flower buds (**Figure 4A**) and anthers from flowers at anthesis (**Figure 4B**) compared to that of CN plants. About ten-fold decrease of the *GLT1* gene was observed in GLT and GLT-RBP lines in the ovary at the flower bud stage (**Figure 4A**) with a significant difference compared to the control CN line. In anthers from flowers at anthesis, downregulation of the *GLT1* was significant in all GLT-RBP and GLT lines analyzed, with reductions up to twelve-fold (GLT-RBP.291) (**Figure 4B**).

### 2.3 Characterization of *CcGLT1* and *CcRBP1* gene downregulation effect on reproductive development of early-flowering Carrizo citrange transformants

**Figure 5:**
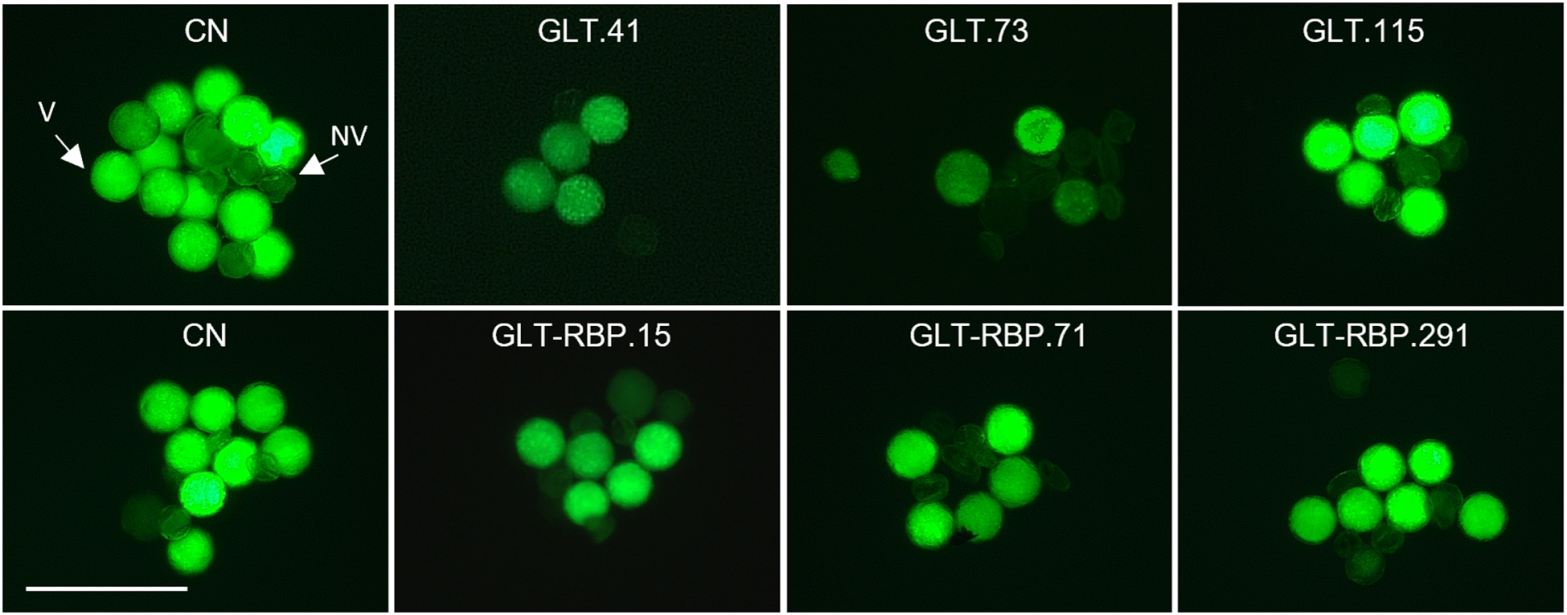
Pollen viability in CN, GLT and GLT-RBP lines performed by FDA assay under fluorescence microscopy. Around 300 pollen grains collected from ten flowers per transgenic line were evaluated. (V) viable pollen, (NV) not viable pollen. Scale bar: 100 µm.

Transgenic plants were characterized by microscopic and histological observations to evaluate male and female performance. Male performance was evaluated by monitoring pollen viability, pollen grain germination and pollen tube growth by *in vitro* culture tests. Results from FDA assays showed a significant reduction of pollen viability in transgenic lines GLT and GLT-RBP compared to the CN control, while no significant differences were observed in RBP lines, suggesting that *RBP1* downregulation in RBP and GLT-RBP lines was not enough to compromise pollen survival (**Figure 5**, **Table 1**).

**Table 1:** Percentage of pollen viability, pollen germinability and pollen tube length in transgenic citrange lines.

Line	Pollen viability (%)	Pollen germinability (%)	Pollen tube length (µm)
CN	70.7 ± 2.5^a^	16.2 ± 0.8^a^	212.0 ± 7.5
GLT.41	60.8 ± 1.1^b^	7.5 ± 1.1^cd^	220.0 ± 13.2
GLT.73	19.9 ± 1.0^e^	7.2 ± 0.1^d^	214.3 ± 8.1
GLT.115	56.7 ± 1.0^b^	12.9 ± 1.0^ab^	220.0 ± 11.0
GLT-RBP.15	40.8 ± 0.8^d^	7.7 ± 1.7^cd^	218.7 ± 6.0
GLT-RBP.71	45.7 ± 2.9^c^	11.0 ± 0.4^bcd^	213.7 ± 4.2
GLT-RBP.291	37.0 ± 2.3^d^	11.7 ± 1.7^bc^	205.0 ± 8.7

Means ± SD followed by different letters indicate significant differences (*p*-value < 0.01) by Tukey’s multiple range test. Pollen viability, germinability and tube length were evaluated from around 300 pollen grains collected from ten flowers per transgenic line.

Similar results were obtained for pollen germination rate *in vitro*, with a significant reduction of the percentage of pollen germination specifically in transgenic lines GLT.41 and GLT.73 and all GLT-RBP lines compared to the CN (**Table 1**), consistent with pollen viability results. However, no difference was found in the pollen tube length from transgenic lines, independently of their genetic background (**Table 1**).

Given the reduced pollen viability and germinability detected in some of the ihp lines, cross-tests were conducted on flowers from Clementine mandarin using pollen from CN, one GLT line (GLT.115) and one GLT-RBP line (GLT-RBP.15). Although seed number in Clementine fruit was lower when pollen from GLT and GLT-RBP lines was used, differences were not significant compared to CN pollen, as the number of seeds formed per fruit was high in all cases, varying between 19 and 23 (**Supplementary Figure S4**). These results indicated that pollen viability was not so compromised to preclude fecundation of compatible ovaries from regular citrus flowers and consequent seed development.

**Figure 6:**
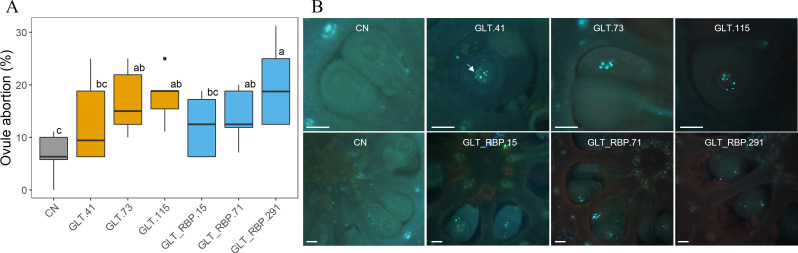
**(A)** Ovule degeneration in self-pollinated pistils from GLT and GLT-RBP transgenic lines. Letters indicate significant difference between ihp transgenic and CN control lines using ANOVA test followed by Tukey *post-hoc* test (*p*-value < 0.01). Around 200 ovules collected from ten flowers per transgenic line were evaluated. **(B)** Ovule degeneration in self-pollinated pistils from CN, GLT and GLT-RBP lines fixed in FFA at 7 days after pollination, monitored on cross sections stained with 0.1% aniline blue in 0.1 N K_3_PO_4_ and observed under a fluorescence microscope. Arrow indicates ovule degeneration fluorescence. Scale bar: 100 µm.

Female performance was assessed by measuring ovule degeneration from self-pollinated pistils stained with aniline blue. Results showed a strong accumulation of callose in the chalazal region from GLT and GLT-RBP transgenic lines, whose fluorescence was observed by aniline blue staining, indicating ovule degeneration with a significant difference compared with CN (**Figures 6A, B**).

**Figure 7:**
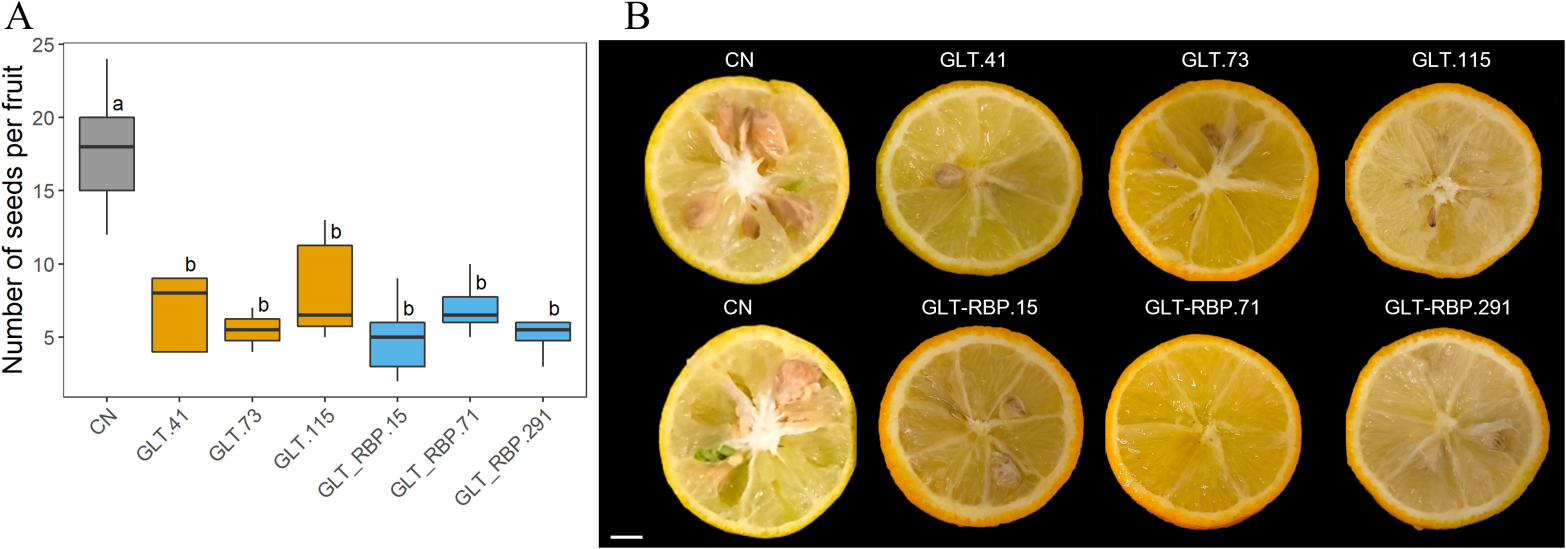
**(A)** Number of seeds collected from transgenic fruits of CN, GLT and GLT-RBP lines. Letters indicate significant difference between transgenics lines using ANOVA test followed by Tukey *post-hoc* test (*p*-value < 0.01). **(B)** Representative picture of open fruits collected from transgenic GLT and GLT-RBP lines compared to CN control. Scale bar: 1 cm.

Seed development was assessed by counting the number of aborted ovules/seeds and regular seeds per fruit. Results showed a significantly reduced number of seeds per fruit in all the GLT and GLT-RBP transgenic lines compared to the CN control (**Figures 7A, B**).

Moreover, some of the ovules/seeds had aborted in GLT and GLT-RBP lines while most of them were fully formed in CN fruits (**Supplementary Table S2**), indicating that seed viability was partially compromised, likely through the action of *GLT1* downregulation, though not completely as some fertilized ovules remained viable to produce regular seeds.

## 3 Discussion

Seedlessness represents one of the most appreciated fruit quality traits in citrus for both fresh and processed markets. Seedless citrus fruits can be obtained by causing or through the random occurrence of spontaneous mutations, female and/or male sterility, embryo abortion, self-incompatibility, application of agronomic techniques (e.g., hormones application), ploidy manipulation, mutation breeding and transgenic approaches (Vardi et al., 2008), most of them associated to parthenocarpy. Male sterility in plants results in the inability to produce functional pollen. In contrast to other fruit crops, male sterility in citrus can be considered a benefit to avoid the presence of seeds, which is one of the most important quality traits for fresh citrus fruit consumption. Among mandarin and mandarin-like cultivars, self-incompatible Clementines have great economic importance and, even though Clementines produce fertile pollen, they bear seedless parthenocarpic fruit due to self-incompatibility. However, the spread of commercial cultivars with sexually inter-compatible pollen is leading to a conspicuous increase in the presence of seeds in seedless genotypes (Distefano et al., 2009). When male sterility is combined with female sterility and parthenocarpy, they ensure obtention of seedless citrus fruits (Yamamoto et al., 1995; Vardi et al., 2008). For example, the combination of male and/or female sterility as well as cross and self-incompatibility with parthenocarpy, widely spread among varieties of commercial interest such as Navel oranges and Clementine mandarins, allows to obtain seedless fruits. The molecular mechanisms underlying male and/or female sterility and seed development in citrus are poorly understood and no specific genes have been proposed as markers and used so far to obtain seedless citrus varieties.

The characterization of candidate genes related to traits of interest as well as the selection of varieties in genetic improvement programs is severely limited by the complex genetics and reproductive biology of *Citrus* species that include long juvenile period, high heterozygosity, cross and self-incompatibility and facultative apomixis. Genetic engineering and the availability of next-generation sequencing technologies allow to accelerate the selection in citrus breeding programs and genetic studies. In this work, an early flowering phenotype was induced in transgenic plants of Carrizo citrange to allow studying the likely involvement of two genes from *C. clementina* in reproductive development through silencing them by RNAi to attempt triggering sterility.

The first studied gene, *CcGLT1*, encodes a protein highly homologous to plant Glycosyltransferase 92 and contains a conserved motif characteristic of this family. Besides, plant orthologs show a common phylogenetic origin with not too much divergence, envisaging a somewhat conserved function. However, the functional analysis of this gene has only been reported in *Arabidopsis*, in which it has been described to be involved in plant reproduction. Several studies performed in *Arabidopsis* revealed that defects in glycosyltransferase-like protein genes (AT3G08550) induce sterility (Cheng et al., 2000; Brocard-Gifford et al., 2004; Kong et al., 2012) and anomalies in ABA metabolism (Brocard-Gifford et al., 2004) whose function in citrus fruit development has been documented (Garcia-Papi and Garcia-Martinez, 1984). According to TAIR database, an *Arabidopsis thaliana* ortholog is highly expressed in plant reproductive tissues and, concordantly with its envisaged function, *in silico* analysis on the citrus transcriptome database revealed high *CcGLT1* transcription level in ovary tissue. Analysis of the 2-kb upstream region of *GLT1* gene was found to contain *cis*-acting elements involved in pollen expression showing a high degree of conservation in sweet orange, Clementine and trifoliate orange, although no conserved pattern in motifs distribution was observed among other species indicating a possible divergence in transcription profiles. Further analysis of *CcGLT1* transcription profile by qRT-PCR revealed an increased accumulation of transcripts in the ovary at the bud stage, and beyond that, in the anthers from flowers at anthesis, supporting the involvement of this gene in reproductive function.

The second characterized gene, *CcRBP1*, encodes a protein highly homologous to RNA binding (RRM/RBP/RNP) proteins and contains all conserved motifs characteristic of this family, besides a clear phylogenetic relationship. This class of ribonucleoproteins (RBPs) play a key role in the regulation of gene expression by controlling the post-transcriptional fate of mRNA (Iadevaia and Gerber, 2015) from genes that seem to be involved in seed development (Kourmpetli et al., 2013), pollen germination and tube growth (Wang et al., 2008) in *Arabidopsis thaliana* and in cytoplasmic male sterility (CMS) in *Brassica rapa* and in cybrid pummelo (Zheng et al., 2014; Jeong et al., 2017). Also, several studies reported in *Arabidopsis* the involvement of a suite of RBPs (nuclear CCCH-type zinc-finger proteins HUA1 and HUA2) in flower organ identity (Rodríguez-Cazorla et al., 2015, Rodríguez-Cazorla et al., 2018) and a severely reduced fertility in mutant plants by their loss-of-function (Rodríguez-Cazorla et al., 2015). As for *CcGLT1*, the promoter sequence of the *CcRBP1* gene was found to contain *cis*-acting elements involved in pollen and embryo-specific expression showing a high degree of conservation in Clementine, sweet and trifoliate oranges and a lack of a specific pattern of *cis*-acting elements distribution in other plants. The pollen and embryo expression of *CcRBP1* was confirmed by qRT-PCR analysis which revealed an increased accumulation of *RBP1* transcripts in the ovary at the bud stage and in anthers from flowers at anthesis.

As both selected genes could influence seed development, three types of transgenic lines were generated, aiming to silence by RNAi (i) the *GLT1*, (ii) the *RBP1* and (iii) both *GLT1-RBP1* genes. Carrizo citrange transgenic plants also contained an *FT* overexpression cassette, that allows early characterization of fruit features (Pons et al., 2014). All transgenic plants showed a clearly visible early flowering phenotype from the early stages of regeneration *in vitro* confirming the overexpression of the *FT* cassette.

All transgenic plants, CN, GLT, RBP and GLT-RBP lines, showed alterations in the architecture as consequence of *FT* overexpression. Because previous studies have reported a reduction in plant size due to the ectopic overexpression of flowering time-related genes in transgenic citrus plants (Peña et al., 2001; Pons et al., 2014), transgenic plants of similar size were chosen in this study to perform the phenotypic characterizations. Even though this selection, RBP and GLT- RBP lines showed stronger alterations on plant growth and development, with significant reductions of internode length and in the number of developed flowers and fruits, envisaging that *CcRBP1* gene is probably more related to the performance of other organs/structures. This later fact precluded the characterization of RBP function on ovule/seed development, because enough material was not obtained to make reliable measurements leaving its role in fertility regulation unresolved. Moreover, the observed detrimental effects on plant development of *RBP1* silencing disabled its potential use as biotechnological target to obtain seedlessness fruits.

In GLT1 and GLT1-RBP transgenic lines, transcription of the *GLT1* gene was significantly downregulated (almost 90% compared to the control CN) in ovaries from flower buds, while in anthers silencing of the *GLT1* gene was observed in flowers at anthesis with a significant difference in GLT and GLT-RBP lines compared to CN. Histological and microscopic observations performed on GLT and GLT-RBP transgenic lines showed that *GLT1* silencing affects pollen performance by reducing pollen viability and germinability. Similarly, a reduction of female performance was observed in ovules from self-pollinated transgenic plants by an increase in the rate of ovule abortion in all GLT and GLT-RBP lines compared with the CN. These reductions in pollen and ovule performances in *GLT1*-silenced lines led to a reduction in the number of seeds in fruits collected from the self-pollinated transgenic plants. However, despite the reduced pollen viability and germination from transgenic plants, the pollen was still able to fertilize non-transgenic Clementine mandarin flowers resulting in the development of seeds in fruits to the same extent than that induced using CN pollen. These results indicate that, to get male sterility, reductions of pollen viability/germinability at least higher than 50% are required. The *GLT1* gene was not widely characterized before in plants, the only reported studies were conducted in *Arabidopsis* in which defective mutants had shown sterility (Cheng et al., 2000; Lertpiriyapong and Sung, 2003; Brocard-Gifford et al., 2004; Kong et al., 2012; Pagant et al., 2013), or male sterility specifically (Brocard-Gifford et al., 2004). Here, the effect of *GLT* gene silencing on male fertility was confirmed by the significant decrease of pollen viability and germinability. Moreover, the drastic reduction in the number of seeds formed in fruit from self-pollinated transgenic plants can be additionally attributed to a reduction in ovule fertility due to *GLT1* silencing, which caused an increase in ovule degeneration. Overall, although the *GLT* silencing did not result in complete male and female sterility, these anomalies contributed to reduce to 28% the number of seeds in fruits from the self-pollinated transgenic plants. Besides effects on reproduction, lethal anomalies in plant growth and development were reported as an effect of *GLT1* silencing in *Arabidopsis* (Cheng et al., 2000; Lertpiriyapong and Sung, 2003; Brocard-Gifford et al., 2004; Pagant et al., 2013). Here, we did not observe growth abnormalities in GLT lines attributable to gene silencing but only those related to *CsFT* overexpression, which were similar in GLT and CN lines. The differences between *GLT1* function in *Citrus* and *Arabidopsis* vegetative development is not surprising as their phylogenetic relationship is clear (involvement in reproduction), but they have diverged along evolution, as it is reflected at least by the low conservation of *cis*-acting elements distribution in their promoter sequences.

GLTs are crucial for plant reproductive viability by contributing to starch and sugar metabolism creating glycans needed for pollen and ovule viability as highlighted by ortholog studies performed in various plant species. Components of the mitogen-activated protein kinase (MAPK) pathway have been associated with reproductive development in other plant species and may interact with GLT-related processes. RNAi-mediated suppression of the *Mitogen-activated protein kinase 4* gene (*SlMPK4*) in tomato caused defects in pollen development leading to lacked viability with alteration of the expression of genes controlling starch and sucrose metabolism including GLTs (Wang et al., 2022). Similarly, a comparative transcriptome analysis of a seedless Ponkan mandarin mutant with its seeded progenitor showed that the upregulated genes in the seedless mutant were mainly related to starch and sucrose metabolism as well as to the MAPK signaling pathway, suggesting that these metabolic processes may be involved in pollen abortion in the mutant (Ye et al., 2020). To the best of our knowledge, alteration on starch and sugar contents during pollen development results in reduction of pollen performance in Clementine (Bennici et al., 2019). GLTs may also indirectly influence downstream pathways, such as the establishment of a GABA gradient in the transmitting tract, which provides positional guidance cues for pollen tube growth and pollen-pistil interaction (Robichaux and Wallace, 2021) although such interactions have not been demonstrated in citrus.

Overall, these results demonstrate the role of the *GLT1* gene in the regulation of reproductive function. Further experiments to knock-out *via* genome editing could be conducted to validate the role of the *GLT1* gene into male and female sterility and to get more efficient seed abortion in citrus fruit.

## 4 Conclusion

Our results indicate that silencing or knock-down of *GLT1* by genetic engineering may contribute to the development of citrus seedless cultivars. Reduction or abolishment of its expression either alone or in combination with that from other target genes involved in ovule or seed development could lead to the generation of fruits completely devoid of seeds. In addition, once the function and the expression pattern of this gene in reproductive tissues are characterized, it opens the way to further functional characterization in other citrus types and other plants for which seedlessness is a highly demanded attribute. Moreover, *GLT1* high expression level in anthers at anthesis and in ovaries at the bud stage makes its 0.7-kb upstream region a promising tool to attempt developing a reproductive tissue-specific promoter to drive the expression of target genes specifically to these tissues, as this region upstream of the transcription start site concentrates all the reproductive tissue-specific motifs. Overall, these results contribute to increase insights into male and female sterility in citrus for the genetic improvement of commercial varieties with seedless fruits.

## 5 Materials and methods

### 5.1 Identification of citrus candidate genes

Genome databases from proprietary seedless and seeded varieties [irradiated mandarin (*Citrus reticulata* Blanco) versus non-irradiated counterparts] were used to select candidate genes affected by SNPs in their genomic sequence. Also, *in silico* gene expression analysis was performed to evaluate pollen and/or ovule/seed tissue distribution of candidate genes according to a citrus transcriptome database (Citrus Annotation Project: http://citrus.hzau.edu.cn/). Sequence similarity searches to candidate genes *CcGLT1* (Gene ID: 18032927) and *CcRBP1* (Gene ID: 18034564) were conducted using the BLASTp program on NCBI database. To explore the phylogenic relationships protein sequences of the candidate genes and orthologs were obtained from Phytozome v13 database (https://phytozome-next.jgi.doe.gov/). Protein sequences (XP_006421212.1 and XP_024035447.1) were submitted to the InterPro database (http://www.ebi.ac.uk/interpro/) to determine their belonging to a characterized family and/or the presence of functionally important domains and sites. The amino acid sequences were aligned using muscle alignment tool and phylogenetic trees were created through MEGA 11 program (Tamura et al., 2021) with the neighbor-joining (NJ) method and bootstrap was set at 1000 replications. The upstream regulatory regions (2.0-kb) of the candidate genes were obtained from Phytozome v13 database and characterized to determine *cis*-acting elements associated with their expression in pollen and/or ovule/seed organs using the New PLACE database (https://www.dna.affrc.go.jp/PLACE/?action=newplace). The results were submitted to the online Gene Structure Display Serve (GSDS, http://gsds.cbi.pku.edu.cn/) for visualization.

### 5.2 qRT-PCR analysis

Expression of candidate genes *CcGLT1* and *CcRBP1* was assayed by quantitative reverse transcription PCR (qRT-PCR) analysis in different tissues from Carrizo citrange (*Citrus sinensis* L. Osb. x *Poncirus trifoliata* L. Raf.) plants including leaves at two developmental stages, ovaries and anthers from flower buds, flowers at pre-anthesis and anthesis and 2-week-old (after anthesis) 5-mm long young fruits (**Figure 1A**). Plant materials were collected from trees held in the germplasm collection at IVIA, in Moncada, Valencia, Spain. Collected samples were immediately frozen in liquid nitrogen, ground to a fine powder and stored at -80°C until further use. Total RNA was extracted from samples and treated with rDNase RNase-free using Nucleo Spin^®^ RNA Plant kit (Macherey-Nagel, Germany). Total RNA was quantified using a NanoDrop^®^ND-1000 (NanoDrop products, Thermo Fisher Scientific, United States) spectrophotometer. First-strand cDNA was synthesized from 1 µg of each DNase-treated RNA using oligo(dT)18 and SuperScriptTM II Reverse Transcriptase (Invitrogen, Thermo Fisher Scientific, United States) according to the manufacturer’s instructions. qRT-PCR analysis was run on QuantStudio™ 3 Real-Time PCR System (Thermo Fisher Scientific, United States) on 500 ng of total cDNA adding 6 µL of SYBR Green PCR Master Mix (Applied Biosystems, USA) and 0.3 µM of gene-specific primers in a total volume of 12 µL. The primer pairs used for *GLT1* and *RBP1* expression were designed based on citrus coding sequences from *Citrus clementina* Hort. ex Tan. database available in Phytozome v13 database. Primer sequences are detailed in **Supplementary Table S1**. Citrus *LCY1* (Alquézar et al., 2009) and *UPL7* (Mafra et al., 2012) genes were used as housekeeping reference genes. The reactions were subjected to temperature cycling as follows: 50°C (20 s), 95°C (10 min), then 40 repeats of 95°C (15 s) and 60°C (40 s), followed by 95°C (15 s), 60°C (1 min) and 95°C (15 s). The amplification efficiencies of each primer pair and the dynamic range of all the genes analyzed were determined by monitoring the variation of ΔCT by using a 10-fold dilution series of a mix of cDNA samples from different tissues as a standard curve. To demonstrate the expression stability of the reference genes *LCY1* and *UPL7* under our experimental conditions, the algorithm geNorm was used (https://genorm.cmgg.be) (Vandesompele et al., 2002). The relative expression level of the target genes normalized to the expression of the housekeeping genes (*LCY1* and *UPL7*) was calculated following the mathematical model described by Livak and Schmittgen (2001). The values reported are the mean ± SD of at least three independent assays. Statistical analyses were performed using ANOVA (*p* < 0.01).

### 5.3 Constructs generation

Using GoldenBraid cloning system v3.0, three RNAi vectors were generated to (i) silence the expression of the *CcGLT1* gene, (ii) silence the expression of the *CcRBP1* gene and (iii) to silence the expression of both *CcGLT1* and *CcRBP1* genes. A schematic representation of the T-DNA region of the constructs used in this work is shown in **Figure 3A**. The 404-bp fragment corresponding to the sequence used in the ihp *CcGLT*i (nucleotide positions 83–486 of the 6084-bp *CcGLT1* gene sequence) was PCR-amplified from a gBlocks Gene Fragment (IDT) using primers B433 and B434 for sense orientation and primers B437 and B438 for antisense orientation. The 415-bp fragment corresponding to the sequence used in the ihp *CcRBP*i (nucleotide positions 3914–4328 of the 8391-bp *CcRBP1* gene sequence) was PCR-amplified from a gBlocks Gene Fragment (IDT) using primers B409 and B427 for sense orientation and primers B439 and B440 for antisense orientation. The 819-bp fragment corresponding to the sequence used in the ihp *CcGLT*i*-CcRBP*i (nucleotide positions 83–486 and 3914–4328 of the 6084-bp *CcGLT1* and 8391-bp *CcRBP1* genes sequences, respectively) was PCR-amplified from a gBlocks Gene Fragment (IDT) using primers B433 and B427 for sense orientation and primers B439 and B438 for antisense orientation (**Supplementary Table S1**). PCR products were gel-purified using an E.Z.N.A. Cycle pure kit (Omega, Norcross, United States). The pUPD2 plasmid and 40 ng of each purified PCR amplicon were used to perform a *Bsm*BI GoldenBraid (GB) reaction as described by Sarrion-Perdigones et al. (2013). pUPD2 sense and pUPD2 antisense parts for each ihp cassette were subjected to further GB reaction with *Bsa*I and assembled into the pDGB3-α2 vector separated by an intron (GB01281) and under the control of the CaMV 35S promoter (GB0552) and the *nos* (nopaline synthase) terminator (GB0037). Sequences of GB-Parts are accessible at GB cloning website (https://goldenbraidpro.com/) using the GB database ID. The ihp cassettes were excised from pDGB3-α2 by digestion with *Hind*III (New England Biolabs) and ligated in the binary vector pROKII-*CsFT* (Pons et al., 2014) digested with the same restriction enzyme and dephosphorylated, to obtain the final pROKII-*CsFT*-*CcGLT*i, pROKII-*CsFT*-*CcRBP*i and pROKII-*CsFT*-*CcGLT*i-*CcRBP*i RNAi plasmids (**Figure 3A**). The empty plasmid pROKII-*CsFT* was used to transform control plants (**Figure 3A**). After confirmation by plasmid restriction analysis and by sequencing, each vector was transferred to *A. tumefaciens* strain EHA105 by thermal shock. T4 DNA ligase was purchased from Promega, *Bsa*I from New England Biolabs, and *BsmB*I from Fermentas. Plasmid extractions were made by using The E.Z.N.A. Plasmid Mini Kit I (Omega Bio-Tek). *Escherichia coli* XL1-Blue strain was used for gene cloning.

### 5.4 Generation of transgenic plants

Epicotyl explants from five-week-old Carrizo citrange *in vitro*-grown seedlings were used for transformation as described in Peña et al. (2004). Briefly, epicotyl explants about 1 cm in length were incubated for 5 min in the bacterial suspension. After removing bacterial debris and drying, explants were transferred to a solid co-cultivation medium [4.4 g/L MS salts (Murashige and Skoog, 1962), 3% (w/v) sucrose, 100 mg/L myo-inositol, 1mg/L nicotinic acid, 1 mg/L pyridoxine hydrochloride, 0.2 mg/L thiamine hydrochloride, 2 mg/L indole-3-acetic acid (IAA), 1 mg/L 2-isopentenyl-adenine (2-ip), 2 mg/L 2,4-dichlorophenoxyacetic acid (2,4-D) and 8 g/L agar, pH 5.7] and maintained for 3 days in the semi-dark at 25°C. Then, the explants were transferred to solid selection medium [4.4 g/L MS salts, 3% (w/v) sucrose, 100 mg/L myo-inositol, 1mg/L nicotinic acid, 1 mg/L pyridoxine hydrochloride, 0.2 mg/L thiamine hydrochloride, 3 mg/L BAP, 8 g/L agar, pH 5.7] supplemented with 100 μg/L kanamycin for nptII selection and 250 μg/L of vancomycin and 500 μg/L of cefotaxime to control bacterial growth. The plates were maintained in the dark at 25°C for two weeks and then transferred to 16 h photoperiod at 25°C. After four-five weeks, regenerated shoots were shoot-tip grafted *in vitro* onto Carrizo citrange seedlings as described in Peña et al. (2008). Seeds used for seedling production came from mother seed trees located at the IVIA germplasm bank in Moncada, Valencia, Spain.

### 5.5 Molecular analysis of the transgenic plants

For molecular analysis, genomic DNA from regenerated plantlets was extracted using the CTAB method as described by McGarvey and Kaper (1991). The presence of the T-DNA was checked by PCR using pairs of specific primers for the different RNAi cassettes (**Supplementary Table S1**). For CsFT, we amplified the region encompassing the end of the 35S promoter (35Sfinal-F) and the entire *CsFT* transgene (FTcs2) to avoid non-specific amplification of the endogenous *FT* gene. Similarly, for *CcGLT1* and *CcRBP1* we used primer designed on the 35S promoter (35Sfinal-F) and the intron (B406 for pROKII-CsFT-CcGLTi and B427 for pROKII-CsFT-CcRBPi, and pROKII-CsFT-CcGLR-CcRBPi). Approximately 2 months after grafting *in vitro*, the PCR-positive plantlets were grafted in the greenhouse onto rough lemon (*C. jambhiri* Lush.) rootstocks. PCR was performed using FIREPol^®^ DNA Polymerase following the manufacturer’s instructions. Reactions for *CsFT* and *nptII* amplification were carried out under the following conditions: 95°C for 5 min, 35 cycles of 95°C for 30 s, 57°C for 30 s and 72°C for 45 sec, followed by 72°C for 10 min. Reactions for ihp constructs amplification were carried out using conditions of 95°C for 5 min, 40 cycles of 95°C for 30 s, 60°C for 30 s and 72°C for 1.5 m, followed by 72°C for 10 min. PCR products were detected by electrophoresis on 1% agarose gels. Silencing of the *CcGLT1* and *CcRBP1* genes in male and female reproductive tissues of the transgenic plants was assayed by qRT-PCR analysis as detailed above. Primer sequences used are indicated in **Supplementary Table S1**.

### 5.6 Histology and microscopic observations

Histological observations were performed to evaluate pollen grain germination, pollen tube growth, and ovule degeneration by *in vitro* culture tests. A minimum of ten flowers per transgenic line was collected. Anthers were removed from the flowers at pre-anthesis and were dried in Petri dishes over silica gel at room temperature. Then, pollen from dehiscent anthers was used for *in vitro* analysis and histological observations. Pollen germination was evaluated as described by Bennici et al. (2019). Pollen viability was assessed by fluorescein diacetate (FDA) staining as described by Heslop-Harrison and Heslop-Harrison (1970). Around 300 pollen grains for each flower were manually tagged on random images. Pollen with bright green fluorescence was classified as viable, while pollen with diminished fluorescence was labeled as dead.

Cross-tests were conducted on flowers from Clementine mandarin to test pollen performance. A batch of 30 flowers randomly selected from the three Clementine plants grown in a greenhouse, located at the IBMCP, in Valencia, Spain, were cross-pollinated one day before anthesis. Flowers from Clementine plants (ten flowers per line, one Clementine plant per line) were emasculated, hand-pollinated with a small paint brush with pollen from CN, GLT.115 and GLT-RBP.15 plants and bagged in cotton tissue. Fruits form cross-pollinated flowers were collected at maturity and evaluated for seed content.

Ovule degeneration was evaluated from self-pollinated transgenic plants. Ten flowers per transgenic line was collected. Hence, anthers were removed from the flowers at pre-anthesis and were dried in Petri dishes over silica gel at room temperature. Then, dehiscent anthers were used to pollinate the emasculated flowers at anthesis of the transgenic plants. The pollinated flowers were bagged to avoid any undesired cross-pollination. The pistils from the self-pollinated flowers were collected after 7 days and fixed in FAA solution (formalin, glacial acetic acid, 70% ethanol, 1:1:18, v/v) (Johansen, 1940) and stored at 4°C until the histological and microscopic observations. The pistils fixed in FAA were washed three times in distilled water and sliced into cross sections as described by Montalt et al. (2019). Then, slices were stained with 0.1% aniline blue in 0.1 N K_3_PO_4_. Around 200 ovules from ten flowers per transgenic line were evaluated. Microscopic observations were performed under a Leica DM5000B microscope (Leica Wetzlar, Germany) by bright-field and fluorescence microscopy for both pollen germination and pollen tube growth and both pollen viability and ovule degeneration evaluation, respectively. Pollen viability was assessed by FDA staining using excitation and emission wavelengths of 494 and 530 nm, respectively. Callose depositions for ovule degeneration evaluation were evaluated using the excitation wavelength of 370 nm and the emission maximum of 509 nm. Images were acquired with a Leica DFC 550 digital camera and analyzed using ImageJ software (https://imagej.nih.gov/). Seed development was assayed by counting the number of seeds in 8 to 21 fruits per plant at the full-colored stage.

## Data Availability

The original contributions presented in the study are included in the article/**Supplementary Material**. Further inquiries can be directed to the corresponding author/s.

## References

[B1] Aleza P. Cuenca J. Juárez J. Navarro L. Ollitrault P. (2016). Inheritance in doubled-diploid clementine and comparative study with SDR unreduced gametes of diploid clementine. Plant Cell Rep. 35, 1573–1586. doi: 10.1007/s00299-016-1972-4, PMID: 27038940

[B2] Aleza P. Juárez J. Cuenca J. Ollitrault P. Navarro L. (2010). Recovery of citrus triploid hybrids by embryo rescue and flow cytometry from 2x × 2x sexual hybridisation and its application to extensive breeding programs. Plant Cell Rep. 29, 1023–1034. doi: 10.1007/s00299-010-0888-7, PMID: 20607244

[B3] Aleza P. Juárez J. Hernández M. Ollitrault P. Navarro L. (2012). Implementation of extensive citrus triploid breeding programs based on 4x × 2x sexual hybridizations. Tree Genet. Genomes 8, 1293–1306. doi: 10.1007/s11295-012-0515-6

[B4] Aloisi I. Distefano G. Antognoni F. Potente G. Parrotta L. Faleri C. . (2020). Temperature-Dependent Compatible and Incompatible Pollen-Style Interactions in Citrus clementina Hort. ex Tan. Show Different Transglutaminase Features and Polyamine Pattern. Front. Plant Sci. 11. doi: 10.3389/fpls.2020.01018, PMID: 32733518 PMC7360793

[B5] Alquézar B. Zacarías L. Rodrigo M. J. (2009). Molecular and functional characterization of a novel chromoplast-specific lycopene β-cyclase from Citrus and its relation to lycopene accumulation. J. Exp. Bot. 60, 1783–1797. doi: 10.1093/jxb/erp048, PMID: 19325166 PMC2671624

[B6] Bennici S. Distefano G. Gentile A. Las Casas G. Di Guardo M. Lana G. . (2019). Temperature stress interferes with male reproductive system development in clementine (Citrus clementina Hort. ex Tan.). Ann. Appl. Biol. 175, 1–13. doi: 10.1111/aab.12508

[B7] Brocard-Gifford I. Lynch T. J. Garcia M. E. Malhotra B. Finkelstein R. R. (2004). The arabidopsis thaliana ABSCISIC ACID - INSENSITIVE8 locus encodes a novel protein mediating abscisic acid and sugar responses essential for growth. Plant Cell 16, 406–421. doi: 10.1105/tpc.018077.1, PMID: 14742875 PMC341913

[B8] Chao C. Fang J. Devanand P. S. (2005). Long distance pollen flow in mandarin orchards determined by AFLP markers –implications for seedless mandarin production. J. Am. Soc Hortic. Sci. 130, 374–380. doi: 10.21273/JASHS.130.3.374

[B9] Cheng J. Lertpiriyapong K. Wang S. Sung Z. R. (2000). The role of the arabidopsis ELD1 gene in cell development and photomorphogenesis in darkness. Plant Physiol. 123, 509–520. doi: 10.1104/pp.123.2.509, PMID: 10859181 PMC59019

[B10] Cheng Z. Song W. Zhang X. (2023). Genic male and female sterility in vegetable crops. Hortic. Sci. 10, uhac232. doi: 10.1093/hr/uhac232, PMID: 36643746 PMC9832880

[B11] Cimen B. Yesiloglu T. Incesu M. Yilmaz B. (2021). Studies on mutation breeding in citrus: Improving seedless types of ‘Kozan’ common orange by gamma irradiation. Sci. Hortic. 278, 109857. doi: 10.1016/j.scienta.2020.109857

[B12] Distefano G. Las Casas G. La Malfa S. Gentile A. Tribulato E. (2009). Pollen tube behavior in different mandarin hybrids. J. Am. Soc Hortic. Sci. 134, 583–588. doi: 10.21273/JASHS.134.6.583

[B13] FAOSTAT (2024). Available online at: https://www.fao.org/faostat/en/ (Accessed March 13, 2025).

[B14] Gambetta G. Gravina A. Fasiolo C. Fornero C. Galiger S. Inzaurralde C. . (2013). Self-incompatibility, parthenocarpy and reduction of seed presence in “Afourer” mandarin. Sci. Hortic. 164, 183–188. doi: 10.1016/j.scienta.2013.09.002

[B15] Garcia-Papi M. Garcia-Martinez J. (1984). Endogenous plant-growth substances content in young fruits of seeded and seedless Clementine mandarin as related to fruit-set and development. Sci. Hortic. 22, 265–274. doi: 10.1016/0304-4238(84)90060-8

[B16] Goldenberg L. Yaniv Y. Porat R. Carmi N. (2014). Effects of gamma-irradiation mutagenesis for induction of seedlessness, on the quality of mandarin fruit. Food Nutr. Sci. 05, 943–952. doi: 10.4236/fns.2014.510105

[B17] Goto S. Yoshioka T. Ohta S. Kita M. Hamada H. Shimizu T. (2016). Segregation and heritability of male sterility in populations derived from progeny of satsuma mandarin. PloS One 11, e0162408. doi: 10.1371/journal.pone.0162408, PMID: 27589237 PMC5010215

[B18] Heslop-Harrison J. Heslop-Harrison Y. (1970). Evaluation of pollen viability by enzymatically induced fluorescence; intracellular hydrolysis of fluorescein diacetate. Stn. Technol. 45, 115–120. doi: 10.3109/10520297009085351, PMID: 4192549

[B19] Huang J. H. Wen S. X. Zhang Y. F. Zhong Q. Z. Yang L. Chen L. S. (2017). Abnormal megagametogenesis results in seedlessness of a polyembryonic ‘Meiguicheng’ orange (Citrus sinensis) mutant created with gamma-rays. Sci. Hortic. 217, 73–83. doi: 10.1016/j.scienta.2017.01.034

[B20] Iadevaia V. Gerber A. P. (2015). Combinatorial control of mRNA fates by RNA-binding proteins and non-coding RNAs. Biomolecules 5, 2207–2222. doi: 10.3390/biom5042207, PMID: 26404389 PMC4693235

[B21] Jeong S.-W. Yi H. Song H. Lee S.-S. Park Y. Hur Y. (2017). Chlorosis of Ogura-CMS Brassica rapa is due to down-regulation of genes for chloroplast proteins. J. Plant Biotechnol. 44, 115–124. doi: 10.5010/JPB.2017.44.2.115

[B22] Johansen D. A. (1940). Plant microtechnique. Ed. Co M.-H. B. (New York: McGraw-Hill Book Company) 523.

[B23] Kong D. Karve R. Willet A. Chen M. Oden J. Shpak E. D. (2012). Regulation of plasmodesmatal permeability and stomatal patterning by the glycosyltransferase-like protein KOBITO1. Plant Physiol. 159, 156–168. doi: 10.1104/pp.112.194563, PMID: 22457425 PMC3406890

[B24] Koura S. Hasegawa K. Yamamoto Y. Yonemoto Y. (2004). Fruit set and fruit growth of seedless cherimoya (Annona cherimola Mill.) induced by GA(3) under greenhouse cultivation in Japan. Proc. Ninth Int. Symp. Plant Brs. Fruit Prod. 653, 63–66. doi: 10.17660/ActaHortic.2004.653.7

[B25] Kourmpetli S. Lee K. Hemsley R. Rossignol P. Papageorgiou T. Drea S. (2013). Bidirectional promoters in seed development and related hormone/stress responses. BMC Plant Biol. 13, 187. doi: 10.1186/1471-2229-13-187, PMID: 24261334 PMC4222868

[B26] Kundu M. Dubey A. (2020). Effect of gamma ray irradiated pollen technique on seed development pattern in citrus. Indian J. Genet. Plant Breed. 80, 450–458. doi: 10.31742/IJGPB.80.4.11

[B27] Lertpiriyapong K. Sung Z. R. (2003). The elongation defective1 mutant of Arabidopsis is impaired in the gene encoding a serine-rich secreted protein. Plant Mol. Biol. 53, 581–595. doi: 10.1023/B:PLAN.0000019067.05185.d6, PMID: 15010620

[B28] Livak K. J. Schmittgen T. D. (2001). Analysis of relative gene expression data using real- time quantitative PCR and the 2-ΔΔCT method. Methods 25, 402–408. doi: 10.1006/meth.2001.1262, PMID: 11846609

[B29] Lora J. Hormaza J. I. Herrero M. Gasser C. S. (2011). Seedless fruits and the disruption of a conserved genetic pathway in angiosperm ovule development. Plant Biol. 108, 5461–5465. doi: 10.1073/pnas.1014514108, PMID: 21402944 PMC3069195

[B30] Mafra V. Kubo K. S. Alves-Ferreira M. Ribeiro-Alves M. Stuart R. M. Boava L. P. . (2012). Reference genes for accurate transcript normalization in citrus genotypes under different experimental conditions. PloS One 7, e31263. doi: 10.1371/journal.pone.0031263, PMID: 22347455 PMC3276578

[B31] McGarvey P. Kaper J. (1991). A simple and rapid method for screening transgenic plants using the PCR. BioFeedback 4, 428–243., PMID: 1793572

[B32] Mesejo C. Martínez-Fuentes A. Reig C. Agustí M. (2008). Gibberellic acid impairs fertilization in clementine mandarin under cross-pollination conditions. Plant Sci. 175, 267–271. doi: 10.1016/j.plantsci.2008.04.008

[B33] Mesejo C. Martínez-Fuentes A. Reig C. Rivas F. Agustí M. (2006). The inhibitory effect of CuSO4 on Citrus pollen germination and pollen tube growth and its application for the production of seedless fruit. Plant Sci. 170, 37–43. doi: 10.1016/j.plantsci.2005.07.023

[B34] Mesejo C. Yuste R. Martínez-Fuentes A. Reig C. Iglesias D. J. Primo-Millo E. . (2013). Self-pollination and parthenocarpic ability in developing ovaries of self-incompatible Clementine mandarins (Citrus clementina). Physiol. Plant 148, 87–96. doi: 10.1111/j.1399-3054.2012.01697.x, PMID: 23002897

[B35] Montalt R. Cuenca J. Vives M. C. Navarro L. Ollitrault P. Aleza P. (2019). Influence of temperature on the progamic phase in Citrus. Environ. Exp. Bot. 166, 103806. doi: 10.1016/j.envexpbot.2019.103806

[B36] Murashige T. Skoog F. (1962). A Revised Medium for Rapid Growth and Bio Assays with Tobacco Tissue Cultures. A. rev. med. Rapid Growth bioass. tob. Tissue cult. Physiol. Plant 15, 473–479. doi: 10.1111/j.1399-3054.1962.tb08052.x

[B37] Pagant S. Bichet A. Sugimoto K. Lerouxel O. Desprez T. McCann M. . (2013). KOBITO1 encodes a novel plasma membrane protein necessary for normal synthesis of cellulose during cell expansion in arabidopsis. Plant Cell 14, 2001–2013. doi: 10.1105/tpc.002873.the, PMID: 12215501 PMC150751

[B38] Pandolfini T. (2009). Seedless fruit production by hormonal regulation of fruit set. Nutrients 1, 168–177. doi: 10.3390/nu1020168, PMID: 22253976 PMC3257607

[B39] Peña L. Cervera M. Fagoaga C. Romero J. Ballester A. Soler N. . (2008). “ Citrus,” in Compendium of transgenic crop plants: tropical and subtropical fruits and nuts. Eds. Kole C. Hall T. C. (Hoboken, NJ: Blackwell Publishing), 1–62.

[B40] Peña L. Martín-Trillo M. Juárez J. Pina J. A. Navarro L. Martínez-Zapater J. M. (2001). Constitutive expression of Arabidopsis LEAFY or APETALA1 genes in citrus reduces their generation time. Nat. Biotechnol. 19, 263–267. doi: 10.1038/85719, PMID: 11231561

[B41] Peña L. Pérez R. M. Cervera M. Juárez J. A. Navarro L. (2004). Early events in agrobacterium-mediated genetic transformation of citrus explants. Ann. Bot. 94, 67–74. doi: 10.1093/aob/mch117, PMID: 15145796 PMC4242373

[B42] Picarella M. E. Mazzucato A. (2019). The occurrence of seedlessness in higher plants; insights on roles and mechanisms of parthenocarpy. Front. Plant Sci. 9. doi: 10.3389/fpls.2018.01997, PMID: 30713546 PMC6345683

[B43] Pons E. Alquézar B. Rodríguez A. Martorell P. Genovés S. Ramón D. . (2014). Metabolic engineering of β-carotene in orange fruit increases its *in vivo* antioxidant properties. Plant Biotechnol. J. 12, 17–27. doi: 10.1111/pbi.12112, PMID: 24034339

[B44] Qin Y. Xu C. Ye Z. Teixeira Da Silva J. A. Hu G. (2015). Seedless mechanism of a new citrus cultivar ‘Huami Wuhegonggan’ (Citrus sinensis × C. reticulata). Pakistan J. Bot. 47, 2369–2378.

[B45] Robichaux K. J. Wallace I. S. (2021). Signaling at Physica Barriers during Pollen–Pistil Interactions. Int. J. Mol. Sci. 22, 12230. doi: 10.3390/ijms222212230, PMID: 34830110 PMC8622735

[B46] Rodríguez-Cazorla E. Ortuño-Miquel S. Candela H. Bailey-Steinitz L. J. Yanofsky M. F. Martínez-Laborda A. . (2018). Ovule identity mediated by pre-mRNA processing in Arabidopsis. PloS Genet. 14, 1–29. doi: 10.1371/journal.pgen.1007182, PMID: 29329291 PMC5785034

[B47] Rodríguez-Cazorla E. Ripoll J. J. Andújar A. Bailey L. J. Martínez-Laborda A. Yanofsky M. F. . (2015). K-homology nuclear ribonucleoproteins regulate floral organ identity and determinacy in arabidopsis. PloS Genet. 11, 1–28. doi: 10.1371/journal.pgen.1004983, PMID: 25658099 PMC4450054

[B48] Sarrion-Perdigones A. Vazquez-Vilar M. Palaci J. Castelijns B. Forment J. Ziarsolo P. . (2013). GoldenBraid 2.0: a comprehensive DNA assembly framework for plant synthetic biology. Plant Physiol. 162, 1618–1631. doi: 10.1104/pp.113.217661, PMID: 23669743 PMC3707536

[B49] Sykes S. R. (2008). Segregation in an ‘Imperial’ mandarin ‘Ellendale’ tangor family for characteristics that contribute to the seedless phenotype. J. Hortic. Sci. Biotechnol. 83, 719–724. doi: 10.1080/14620316.2008.11512450

[B50] Tamura K. Stecher G. Kumar S. (2021). MEGA11: molecular evolutionary genetics analysis version 11. Mol. Biol. Evol. 38, 3022–3027. doi: 10.1093/molbev/msab120, PMID: 33892491 PMC8233496

[B51] Trojak-Goluch A. Kawka-Lipińska M. Wielgusz K. Praczyk M. (2021). Polyploidy in industrial crops: applications and perspectives in plant breeding/agronomy11122574. Agronomy 11, 2574. doi: 10.3390/agronomy11122574

[B52] Vandesompele J. De Preter K. Pattyn F. Poppe B. Van Roy N. De Paepe A. . (2002). Accurate normalization of real-time quantitative RT-PCR data by geometric averaging of multiple internal control genes. Genome Biol. 3, 0034. doi: 10.1186/gb-2002-3-7-research0034, PMID: 12184808 PMC126239

[B53] Vardi A. Levin I. Carmi N. (2008). Induction of seedlessness in citrus : from classical techniques to emerging biotechnological approaches. J. Am. Soc Hortic. Sci. 133, 117–126. doi: 10.21273/JASHS.133.1.117

[B54] Varoquaux F. Blanvillain R. Delseny M. Gallois P. (2000). Less is better: new approaches for seedless fruit production. Trends Biotechnol. 18, 233–242. doi: 10.1016/S0167-7799(00)01448-7, PMID: 10802558

[B55] Wang J. Li M. Zhuo S. Liu Y. Yu X. Mukhtar S. . (2022). Mitogen-activated protein kinase 4 is obligatory for late pollen and early fruit development in tomato. Hortic. Res. 9, uhac048. doi: 10.1093/hr/uhac048, PMID: 35591931 PMC9113226

[B56] Wang Y. Zhang W. Z. Song L. F. Zou J. J. Su Z. Wu W. H. (2008). Transcriptome analyses show changes in gene expression to accompany pollen germination and tube growth in arabidopsis. Plant Physiol. 148, 1201–1211. doi: 10.1104/pp.108.126375, PMID: 18775970 PMC2577266

[B57] Wilcock C. Neiland R. (2002). Pollination failure in plants: why it happens and when it matters. Trends Plant Sci. 7, 270–277. doi: 10.1016/S1360-1385(02)02258-6, PMID: 12049924

[B58] Xiao J. Zhang L. Fan F. Liu X. (2018). Comparative transcript profiling reveals the mechanism of female sterility associated with seedless Ponkan mandarin (Citrus reticulata Blanco). Genome 61, 595–604. doi: 10.1139/gen-2017-0215, PMID: 29958094

[B59] Yamamoto M. Matsumoto R. Yamada Y. (1995). Relationship between sterility and seedlessness in Citrus. Eng. Gkk. zsh. 64, 23–29. doi: 10.2503/jjshs.64.23

[B60] Ye L. X. Gan Z. M. Wang W. F. Ai X. Y. Xie Z. Z. Hu C. G. . (2020). Comparative analysis of the transcriptome, methylome, and metabolome during pollen abortion of a seedless citrus mutant. Plant Mol. Biol. 104, 151–171. doi: 10.1007/s11103-020-01034-7, PMID: 32656674

[B61] Zhang S. Shi Q. Albrecht U. C.OMMAJ.R.X.X.X R. G. S. Stange R. Mccollum G. . (2017). Comparative transcriptome analysis during early fruit development between three seedy citrus genotypes and their seedless mutants. Nat. Publ. Gr. 4, 1–12. doi: 10.1038/hortres.2017.41, PMID: 28904803 PMC5596110

[B62] Zheng B. Fang Y. Pan Z. Sun L. Deng X. Grosser J. . (2014). iTRAQbased quantitative proteomics analysis revealed alterations of carbohydrate metabolism pathways and mitochondrial proteins in a male sterile cybrid pummelo. J. Proteome Res. 13, 2998–3015. doi: 10.1021/pr500126g, PMID: 24824475

[B63] Zheng B. Wu X. Ge X. Deng X. Grosser J. W. Guo W. (2012). Comparative transcript profiling of a male sterile cybrid pummelo and its fertile type revealed altered gene expression related to flower development. PloS One 7, 1–13. doi: 10.1371/journal.pone.0043758, PMID: 22952758 PMC3429507

