# Supplementary material for: Silencing of a glycosyltransferase-like protein in citrus reduces male and female fertility impacting seed development in self-pollinated fruit
Source: Front Plant Sci. 2026 Jan 21;16:1629727. doi: 10.3389/fpls.2025.1629727 (PMC12868143; doi:10.3389/fpls.2025.1629727)
Supplement: Supplementary file 1 [file DataSheet1.docx]

Supplementary Material


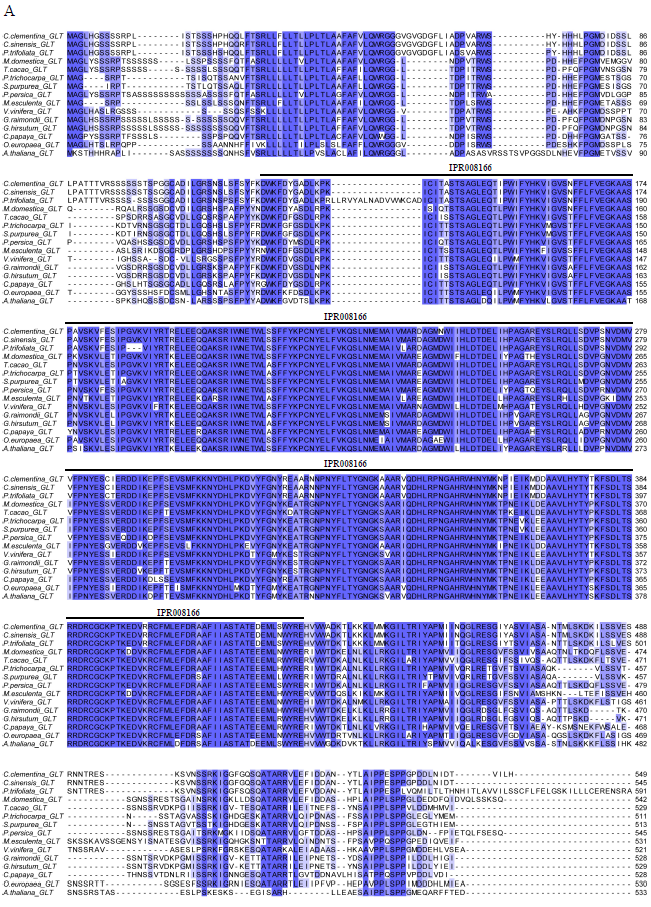


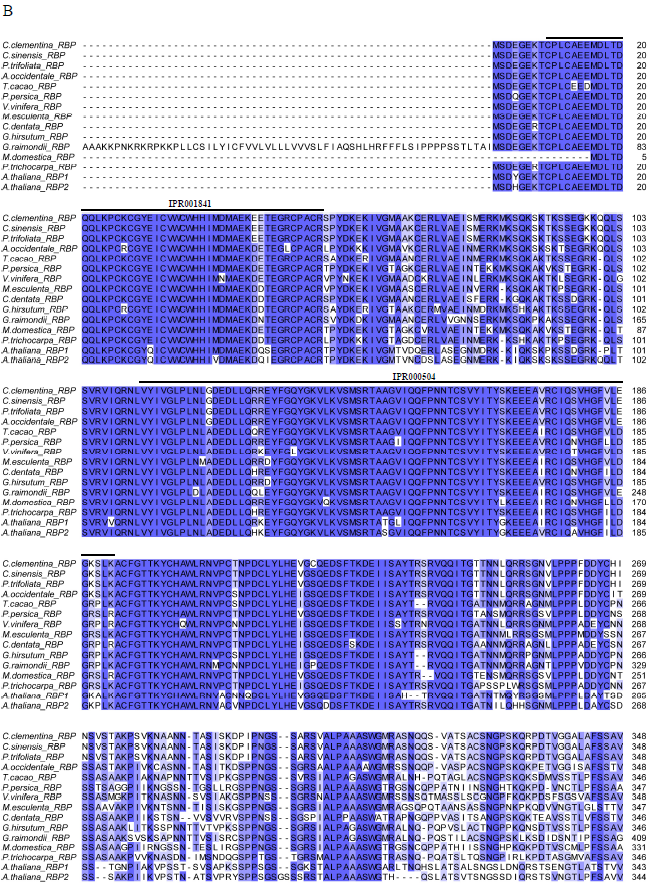

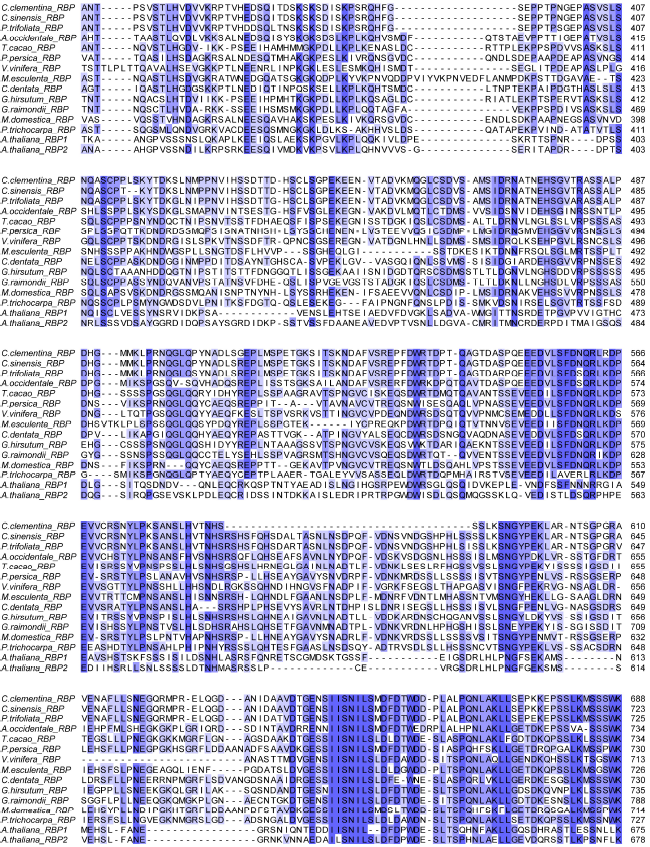

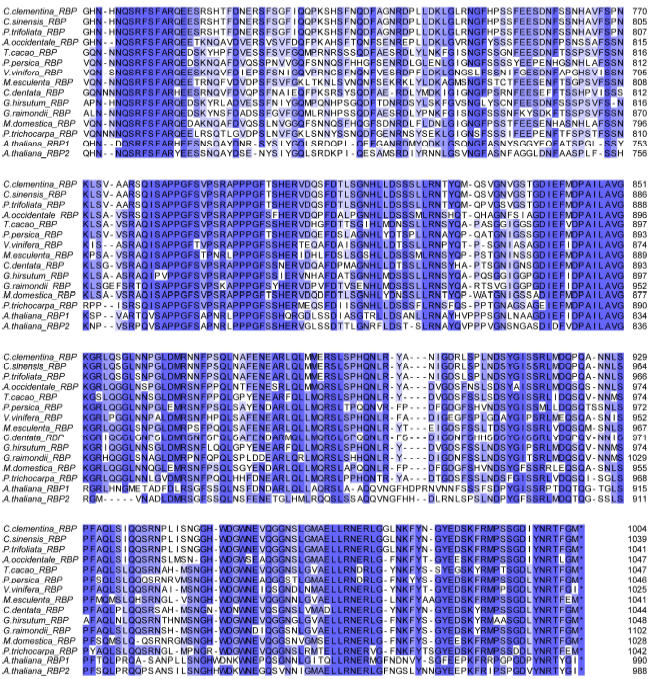


**Supplementary Figure S1. (A)** Amino acid sequence alignment of glycosyltransferase-like proteins (GLT1) from *Citrus clementina* with 14 different GLT proteins from other plants. Characteristic Glycosyltransferase family 92 domain motif (IPR008166) present in GLT proteins is indicated. **(B)** Amino acid sequence alignment of RNA binding (RRM/RBD/RNP motifs) family proteins (RBP1) from *Citrus clementina* with 14 different RBP proteins from other plants. Characteristic RNA recognition motif domain (IPR000504) and Zinc finger, RING-type (IPR001841) present in RBP proteins are indicated. The alignments were generated with Clustal Omega and Jalview v.2.11.2.6. Amino acids are color coded according to their conservancy: in dark violet are fully conserved, while those marked in light gray violet present less similarity.


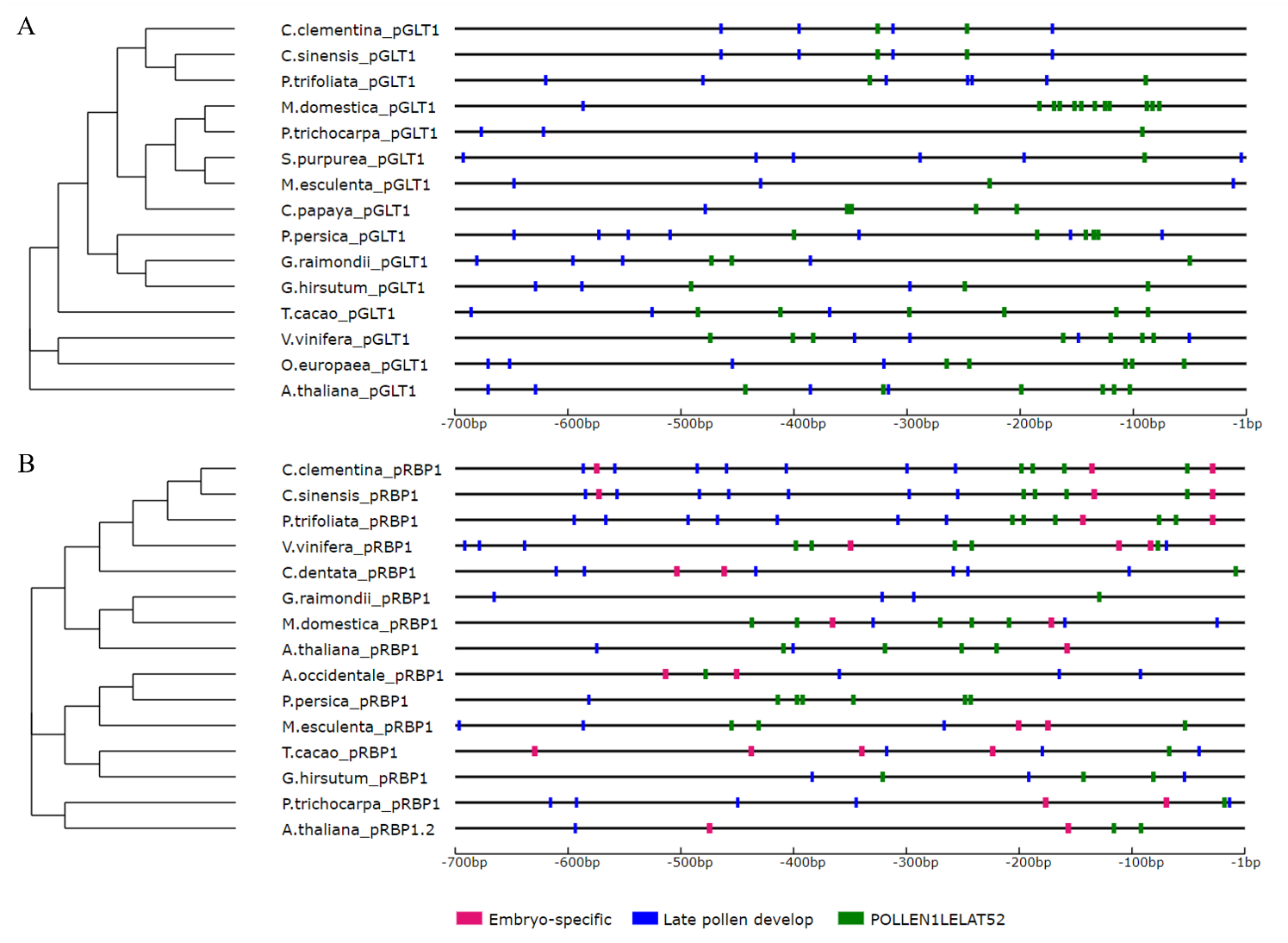


**Supplementary Figure S2**. **(A)** Phylogenetic tree of promoter region (2kb) from glycosyltransferase-like proteins (GLT1) encoded by *Citrus clementina* (Ciclev10004681m.g), *Citrus sinensis* (Cs_ont_9g017790.1), *Poncirus trifoliata* (Ptrif.0009s1448), *Gossypium raimondii* (Gorai.007G355500), *Gossypium hirsutum* (Gohir.D11G324700), *Malus domestica* (MD12G1085800), *Olea europaea* (Oeu027348.1), *Carica papaya* (evm.TU.supercontig_120.11), *Populus trichocarpa* (Potri.016G041900), *Salix purpurea* (SapurV1A.0018s0900), *Manihot esculenta* (Manes.09G016100), *Theobroma cacao* (Thecc.K0006500), *Vitis vinifera* (VIT_213s0019g01720), *Prunus persica* (Prupe.7G049900), *Arabidopsis thaliana 1* (AT3G08550), *Arabidopsis thaliana 2* (AT3G57200) and *Arabidopsis thaliana 3* (AT2G41451). **(B)** Phylogenetic tree of promoter region (2kb) from RNA binding (RRM/RBD/RNP motifs) family proteins (RBP1) encoded by *Citrus clementina* (Ciclev10027731m.g), *Citrus sinensis* (Cs_ont_8g004030), *Poncirus trifoliata* (Pt8g008970.1), *Vitis vinifera* (VIT_206s0004g03580), *Castanea dentata* (Caden.08G041800), *Gossypium raimondii* (Gorai.004G002000), *Malus domestica* (MD03G1108000), *Arabidopsis thaliana 1* (AT3G45630), *Anacardium occidentale* (Anaoc.0005s1250), *Prunus persica* (Prupe.6G091300), *Manihot esculenta* (Manes.04G026900), *Theobroma cacao* (Thecc.09G336200), *Gossypium hirsutum* (Gohir.A07G093300), *Populus trichocarpa* (Potri.009G026200), and *Arabidopsis thaliana 2* (AT5G60170). The dendrograms were generated with MEGA 11 using the Neighbor-joining method. Structure diagrams of cis-acting elements in the 700-bp upstream of the transcription start site (TSS) of both genes is shown to the right of the phylogenetic tree. Cis-acting elements were analyzed using the New PLACE database. In the *CcGLT1* promoter, the *cis*-acting elements Late pollen develop (GTGA) and POLLEN1LELAT52 (AGAAA), both specific for pollen expression, were found at the same positions than in the corresponding gene of sweet orange (*CcGLT1*) and very close to those of *PtGLT1,* from trifoliate orange **(A)**. Both motifs were also found in sequences of the GLT promoters from other plants, but a reduced degree of conservation was found in relation to their location **(A)**. The same cis-elements, Late pollen develop and POLLEN1LELAT52, were found in the *CcRBP1* promoter with the addition of the Embryo motif (WAACCA), specific for embryo expression **(B)**. Similarly to the *GLT* promoter, a high degree of conservation in the position of the motifs was observed between Clementine, sweet orange and trifoliate orange while no specific pattern was observed among other plant species **(B)**.


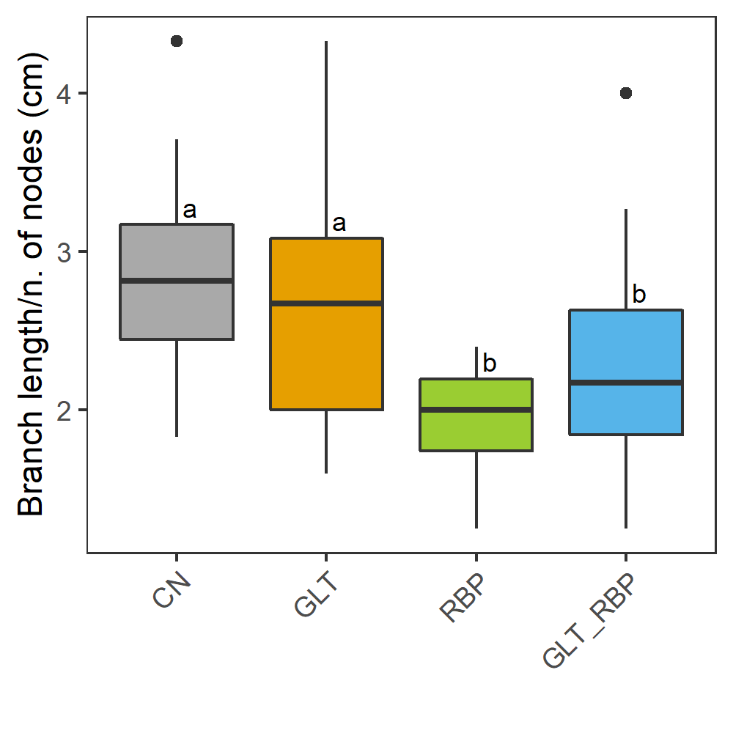


**Supplementary Figure S3.** Plant architecture expressed as branch length/number of nodes of 8 plants from CN, GLT, RBP and GLT-RBP lines. Letters indicate significant difference between transgenic lines using ANOVA test followed by Tukey post-hoc test (*p*-value < 0.01).


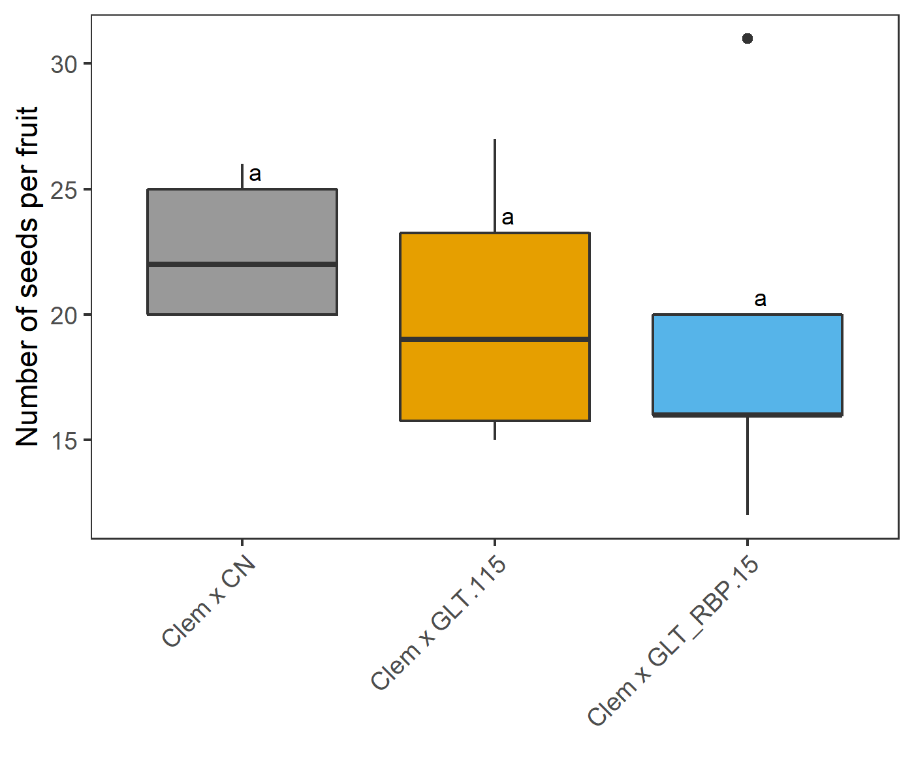


**Supplementary Figure S4.** Number of seeds collected from self-incompatible Clementine mandarin fruits pollinated with pollen from CN, GLT.115 and GLT-RBP.15 lines. Same letter indicates lack of significant differences between lines using ANOVA test followed by Tukey post-hoc test (*p*-value < 0.01). Around 20 fruits per plant at the full-colored stage were evaluated.

**Supplementary Table S1.** Primers used in this study. Nucleotides used to incorporate sequences of interest into Golden Braid system are red-lettered.

**Supplementary Table S2**. Fruit size and diameter, number of normal and aborted seeds, and ovule abortion in mature fruit from transgenic CN, GLT, and GLT-RBP lines.
